# Ketamine’s impact on rumination-related brain dynamics: insights from a randomized controlled fMRI trial

**DOI:** 10.1038/s41398-026-04392-w

**Published:** 2026-08-18

**Authors:** Marvin S. Meiering, David Weigner, Matti Gärtner, Luisa Carstens, Christian Keicher, Gerd Luippold, Andreas Wunder, Anne Weigand, Simone Grimm

**Affiliations:** 1 Institute of Neuroscience and Biopsychology for Clinical Application, Medical School Berlin, Berlin, Germany; 2 Department of Psychology, Freie Universität Berlin, Berlin, Germany; 3 Charité Research Organisation GmbH, Berlin, Germany; 4 Experimental Medicine, Boehringer Ingelheim Pharma GmbH & Co. KG, Biberach an Der Riss, Germany; 5 Free Consultant, Lübeck, Germany; 6 Department of Psychiatry, Psychotherapy and Psychosomatics, University Hospital of Psychiatry, University of Zurich, Zurich, Switzerland

**Keywords:** Neuroscience, Psychology

## Abstract

Rumination has been associated with aberrant dynamics of a coactivation pattern (CAP) comprising the default mode (DMN) and frontoparietal (FPN) networks. Ketamine exerts rapid antidepressant effects and may influence these dynamics via glutamatergic mechanisms. In a randomized, double-blind, placebo-controlled fMRI study, we investigated ketamine’s effects on dynamic CAPs associated with rumination and examined whether inhibition of glutamatergic release through lamotrigine attenuates these effects. Seventy-five healthy adults were randomized to placebo–placebo, placebo–ketamine, or lamotrigine–ketamine treatment. Resting-state fMRI was acquired at baseline, during ketamine/placebo infusion, and 24 h post-infusion. Whole-brain CAP analysis identified seven recurring network configurations. Occurrence rates were examined for group differences, while controlling for age, sex, and drug plasma concentrations. Rumination was assessed using a validated self-report questionnaire. A hybrid DMN + FPN CAP showed a positive association with rumination at baseline. Ketamine acutely reduced the occurrence rate of this hybrid CAP compared to placebo, with larger decreases in individuals reporting higher rumination. These effects were transient, returning to baseline after 24 h. Exploratorily, ketamine also reduced engagement of a canonical somatomotor CAP during infusion. Lamotrigine pretreatment nominally attenuated ketamine-induced changes across analyses. Ketamine transiently alters dynamic brain states implicated in rumination and somatosensory processing, and preliminary evidence suggests partial glutamatergic mediation. These findings provide insights into ketamine’s mechanism of action.

## Introduction

The N-methyl-D-aspartate (NMDA) receptor antagonist ketamine is a rapid acting treatment for patients with (treatment-resistant) major depressive disorder (MDD) and anxiety disorders [1–3]. At the molecular level, ketamine is thought to exert its effects primarily on the glutamatergic system, facilitating processes that ultimately promote neuroplasticity [4–7]. Specifically, subanesthetic doses of ketamine have been demonstrated to induce a transient increase in glutamatergic transmission by blocking NMDA receptors located on GABAergic interneurons and disinhibition of pyramidal neurons. Consequently, α-amino-3-hydroxy-5-methyl-4isoxazolepropionic acid (AMPA) receptors are activated resulting in enhanced levels of brain derived neurotrophic factor (BDNF), facilitating synaptic and dendritic growth within the prefrontal cortex and hippocampus [4, 5]. Despite substantial effort over the past decade, ketamine’s neurofunctional mechanism of action remains poorly understood. One promising approach is the combined administration of ketamine with compounds that inhibit glutamatergic release, such as lamotrigine [8]. As ketamine is thought to enhance glutamatergic neurotransmission, whereas lamotrigine inhibits presynaptic glutamate release, the combined administration of both compounds has been hypothesized to attenuate ketamine-induced glutamatergic effects. Comparing ketamine alone with the combined administration of ketamine and lamotrigine may therefore help identify which of ketamine’s effects are mediated by stimulation of the glutamatergic system. Through this method, several studies have advanced our understanding of ketamine’s mechanism of action in the human brain [9–13]. Moreover, the heterogeneity of MDD might be one potential reason why conclusive evidence of ketamine’s effects is still pending, warranting investigations that elucidate ketamine’s influence on distinct dimensions of psychopathology. One dimension that has stimulated considerable research in the last two decades is repetitive negative thinking (RNT) and its depression-specific facet rumination [14, 15].

Rumination is characterized by the intrusive and repetitive dwelling on past negative experiences or depressed mood [16–18] and has been observed across several mental disorders as well as in healthy populations [14, 15, 19]. Importantly, rumination increases the risk for psychopathology considerably [19–22], thereby posing a crucial transdiagnostic risk factor. On the neurofunctional level, rumination has been characterized as the impaired ability to flexibly recruit large-scale networks in response to contextual demands, potentially reflecting cognitive and attentional control impairments [23–27]. Herein, the Default Mode Network (DMN), salience network (SAL) and frontoparietal network (FPN) are of particular interest. According to Menon’s triple network model, the DMN, FPN and SAL serve complementary functions [28, 29]. The DMN, which is primarily located in medial regions like the medial prefrontal cortex and posterior cingulate cortex, is active at rest and has been associated with self-referential processing [29]. The FPN, on the other hand, is active during cognitively demanding tasks and supports cognitive control and executive processes [30]. It is predominantly anchored in the dorsolateral prefrontal cortex and parietal cortices. Lastly, the SAL, which is primarily located in the dorsal cingulate cortex and anterior insula, is sensitive to changes in the internal or external milieu and orchestrates the recruitment of the DMN and FPN in response to environmental demands [31–34]. Aberrant interactions between the DMN, FPN and SAL have been related to rumination through prolonged network recruitment when contextual demands require flexible and coordinated activation or deactivation of these networks [23–27]. This inability to dynamically shift between the DMN, FPN, and SAL is thought to contribute to the debilitating repetitiveness and excessive internal focus that characterize rumination.

Although rumination has been extensively investigated using static neuroimaging methods [35], recent evidence highlights the non-stationary nature of brain network interactions [36–38]. In this context, dynamic coactivation pattern (CAP) analysis has emerged as a promising method, as it analyzes the rise and fall of brain coactivation configurations at the level of single volumes, thereby providing fine-grained measures of time-dependent network recruitment [38–40]. Herein, rumination has been linked to greater time spent in a hybrid brain state that simultaneously engages task-negative (DMN) and task-positive networks (FPN, SAL) in depressed samples [25, 41, 42]. These findings are corroborated by a recent study in healthy individuals, showing that trait RNT is associated with the persistence of a hybrid network configuration including the DMN and FPN (DMN + FPN) during an RNT induction task [43]. Together, mounting evidence supports that rumination is linked to the occurrence rate and persistence of a hybrid coactivation state that engages functionally antagonistic networks, potentially impairing attentional switching capacities that would facilitate disengagement from rumination.

Ketamine has been shown to effectively reduce rumination [44] and to elicit profound changes in the temporal dynamics of large-scale networks [45]. However, to our knowledge, no study to date has examined how ketamine acutely affects brain network dynamics previously linked to rumination in healthy individuals, nor whether pretreatment with lamotrigine would attenuate these changes. Accordingly, the present study aims to provide valuable insights into ketamine’s mechanism of action by examining rumination-related brain network dynamics and the potential mediating role of the acute glutamatergic surge that follows its administration [4, 5]. Specifically, we predict that the occurrence rate of the hybrid DMN + FPN coactivation pattern is positively associated with rumination at baseline and that ketamine would acutely reduce the occurrence rate of this network configuration. The hybrid DMN + FPN CAP presents a promising target for ketamine’s effects not only because it relates to RNT [25, 41–43], but also because ketamine’s neuroplastic effects are most prominently observed in the prefrontal cortex [4, 5], an area critically engaged in the DMN + FPN CAP [25, 41–43]. Moreover, the hybrid DMN + FPN configuration is hypothesized to respond to ketamine treatment more strongly in individuals reporting higher levels of rumination. To determine whether the neurofunctional effects of ketamine are mediated by the glutamatergic surge following its administration, we predict that inhibiting glutamatergic release through concurrent administration of lamotrigine would reduce ketamine’s effects. Exploratorily, the effects of ketamine on other network dynamic configurations are investigated.

## Methods

### Participants

A total of 75 healthy male and female subjects aged 18–45 years were enrolled. The sample size was determined based on a power analysis for a one-way ANOVA assuming an alpha of 0.05, a beta of 0.8 and a large effect of f = 0.4 [46]. Exclusion criteria were history of or current psychiatric conditions, as determined by the SCID-5-CV at screening, a positive drug screen, previous participation in studies that used experimental paradigms employed in the study, prescribed psychotropic medication within 28 days prior to screening and non-prescription medication within 48 h prior to treatment visit. Further exclusion criteria were a history of relevant neurological diseases, migraine headaches, relevant medical conditions, MRI exclusion criteria, and pregnancy. All participants gave written consent to participate in the study, which was approved by the local ethics committee (Landesamt für Gesundheit und Soziales Berlin / Berlin State Office for Health and Social Affairs and Bundesamt für Arzneimittel und Medizinprodukte / Federal Institute for Drugs and Medical Devices). The study is registered at ClinicalTrials.gov (NCT04156035). No patients were included.

### Study design

**Fig. 1:**
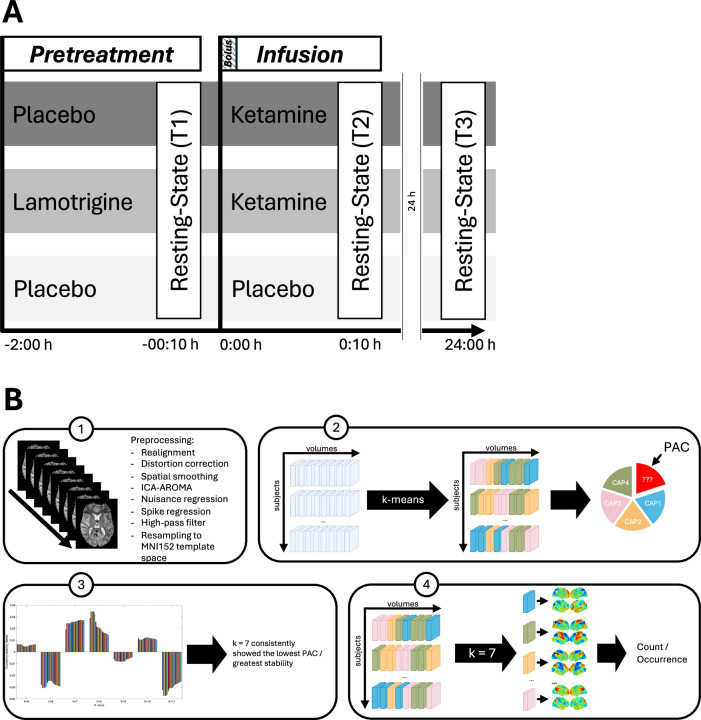
Study design and analytical pipeline. **A** Study design. **B** Analytical pipeline. 1) The fMRI data was preprocessed, denoised and resampled to the MNI152 template space. 2) K-means clustering was performed 50 times using a random subsample comprising 80% of the volumes from all subjects to cluster volumes into a set of recurring coactivation patterns. Proportion of ambiguously clustered pairs was calculated to determine the stability of the clustering solutions. The process was repeated for k = 5 to k = 11 to conclude a winning parameter. 3) PAC was compared across the different k-means iterations to determine the clustering solution with the greatest stability. K = 7 turned out to be the winning parameter with a mean stability of 77.7%. 4) Finally, k-means clustering was performed again – using all volumes from all subjects – to determine the spatial layout of the final CAPs and their counts/occurrences.

Details regarding blinding, randomization, sample size calculation and adverse events can be found in the supplement. Briefly, participants were randomly assigned to one of three treatment groups in a 1:1:1 ratio: pretreatment with placebo followed by a placebo infusion (placebo-placebo group, PP), pretreatment with placebo followed by a ketamine infusion (placebo-ketamine group, PK), or pretreatment with lamotrigine followed by a ketamine infusion (lamotrigine-ketamine group, LK, see Fig. 1A). All participants underwent two scanning sessions on two consecutive days. Pretreatment with an oral dose of 300 mg lamotrigine (LK) or matching placebo (PP, PK) occurred 2 h before the scanning procedures (−2:00 h with respect to infusion onset). Then, participants were intravenously administered racemic ketamine or placebo (0.12 ± 0.003 mg/kg ketamine during the first minute followed by a continuous infusion of 0.31 mg/kg/h for about 55 min). Blood samples were collected −3:00, −1:30, −1:00, −0:30, +0:55, and +2:00 h with respect to infusion onset to determine lamotrigine plasma levels and have been published elsewhere [11]. Plasma ketamine concentrations were collected immediately after the MRI scan ( + 0:55 h from infusion onset). Participants underwent the same scanning procedure without drug treatment 24 h later to investigate potential delayed effects. Notably, the elimination half-life of lamotrigine following a 240 mg dose has been estimated to be approximately 35 h [47]. Administration of 300 mg lamotrigine is therefore expected to result in residual plasma concentrations 24 h post infusion.

Resting-state fMRI was performed at baseline before ketamine administration (T1, see Fig. 1A), during infusion (T2) and 24 h after infusion (T3). Participants were instructed to relax, stay awake, and keep their eyes open to look at a fixation cross. Additional experiments and modalities employed during this study have been reported elsewhere [10–13, 48].

### Psychometric assessments

A subset of subjects participated in an optional follow-up assessment after trial completion to obtain self-report measures of rumination, namely the German version of the Response Styles Questionnaire (RSQ; [49, 50]).

### Image acquisition and analysis

Imaging acquisition parameters are given in the supplement. Brain image preprocessing was carried out using FEAT (FMRI Expert Analysis Tool; [51–53]) version 6, as part of FSL (FMRIB’s Software Library; [54–56]). Detailed information regarding the preprocessing is provided in the supplement and Fig. 1B. Briefly, fMRI volumes were realigned, registered to the MNI152 template including distortion correction and smoothed with a 5 mm FWHM kernel. Then, initial denoising was performed with ICA-AROMA [57, 58], followed by an additional denoising step regressing out residual signals from the white matter, cerebrospinal fluid, global grey matter and 24 motion parameters [59] combined with spike regression.

### Coactivation pattern analysis

We employed a whole-brain, seed-free, voxel-wise dynamic coactivation pattern (CAP) analysis using the TbCAPs Toolbox [39]. The seed-free CAP approach used here has the advantage that all fMRI volumes are included in the process, presumably increasing the reliability of derived dynamic CAP metrics and the detectability of brief network configurations, particularly those involving the DMN [60]. Further, the omission of a large number of volumes following typical CAP analysis (including only an arbitrary number of volumes that exceed coactivation strength with a given seed) essentially breaks the temporal autocorrelation of the data, potentially distorting metrics that rely on volume-to-volume dependencies (e.g. transitional probabilities or persistence). Further, CAP analysis was restrained to grey matter voxels within a grey matter mask of the MNI152 template. Consensus clustering was employed to empirically determine the number of CAPs present in the data. For each number of k clusters considered stable, k-means clustering was run several times using a randomly selected subsample of the original data without replacement [61, 62]. During k-means clustering, each fMRI volume was assigned to one of the prespecified clusters and the assignment was stored in a consensus matrix. Optimal clustering would result in fMRI volumes that are either consistently clustered together or separately over each iteration. Following suggestions from a previous study showing that clustering for k < 5 or k > 11 results in less stable CAPs [40], we performed k-means clustering with k values of 5–11. An optimal clustering solution for k = 7 was determined based on consensus clustering with 50 independent runs for each k, randomly sampling 80% of the original data, using the proportion of ambiguously clustered pairs as cost metric [63] (see Figure S2 and S3). The clustering solution with k = 8 also performed similarly well compared to k = 7. However, visual inspection of the clustering quality across different definitions of stability indicated substantial variability (see step 3 in Fig. 1B or figure S3). Eventually, we opted for k = 7 since it consistently exhibited the greatest stability across different definitions. Then, k-means clustering with k = 7 was run 100 times to bypass local minima and instability of the resulting CAPs, resulting in whole-brain maps that characterize regions that are simultaneously co(de)activated. Afterwards, FSL’s spatial cross-correlation tool [54, 55] was used to determine the spatial similarity between the seven CAP maps and Schaefer’s cortical seven network parcellation to determine correspondences between the CAPs found in our sample and previously defined networks [64]. Finally, the total number of volumes spent in each CAP (count / occurrences) as well as the number of volumes that were consecutively spent in each CAP (persistence) were computed.

### Statistical analyses

Statistical analyses were conducted in R, version 4.3.1 [65]. Counts (occurrences) of the hypothesized DMN + FPN CAP were tested for differences between groups (PK, LK, PP) using permutation testing, with sex and age included as covariates of no interest. Additionally, demeaned ketamine (single time point) and lamotrigine plasma levels (area under the curve) were included in the statistical models as covariates of no interest. Standardized effect sizes are provided as Cohen’s d. To assess the association between CAP occurrences and rumination, linear regression models were employed and tested using permutation testing and bootstrap confidence intervals. In accordance with the severe flaws associated with null-hypothesis significance testing and the *p*-value [66, 67], results of a priori analyses are interpreted given the effect sizes and associated confidence intervals. Accordingly, *p*-values are provided as a complementary statistic for a priori hypotheses but are not interpreted. For exploratory analyses, a hierarchical approach was chosen. First, potential group differences were examined with a permutation-based ANCOVA [68], including age, sex and plasma concentration of ketamine and lamotrigine as covariates of no interest. In case of a significant ANCOVA (p-FDR < 0.05), permutation-based paired comparisons were employed. The code used to generate the results can be requested from the corresponding author.

## Results

A total of 62 subjects remained in the final sample (see flow chart in the supplement). Reasons for exclusion from the fMRI analysis included structural abnormalities and excessive head motion ( > 1.5 mm framewise displacement at any time point during at least one of the three scanning sessions). Average motion among subjects included in the analyses was 0.089 mm (SD = 0.029), with no significant differences between groups (*F*(2, 56) = 0.833, *p* = 0.448). Furthermore, no significant group differences in motion were observed for each time point separately (T1: *F*(2, 56) = 0.695, *p* = 0.508; T2: *F*(2, 56) = 2.400, *p* = 0.102; T3: *F*(2, 56) = 0.086, *p* = 0.918, a figure is provided in the supplement). The optional post hoc questionnaires were completed by 64 subjects from the initial sample, 54 remained for analysis after excluding participants with corrupted fMRI data. No statistically meaningful differences of exclusion rates were found between the groups (*χ*^2^(2) = 2.419, *p*_*Fishers-exact*_ = 0.401).

### Coactivation pattern analysis

**Fig. 2:**
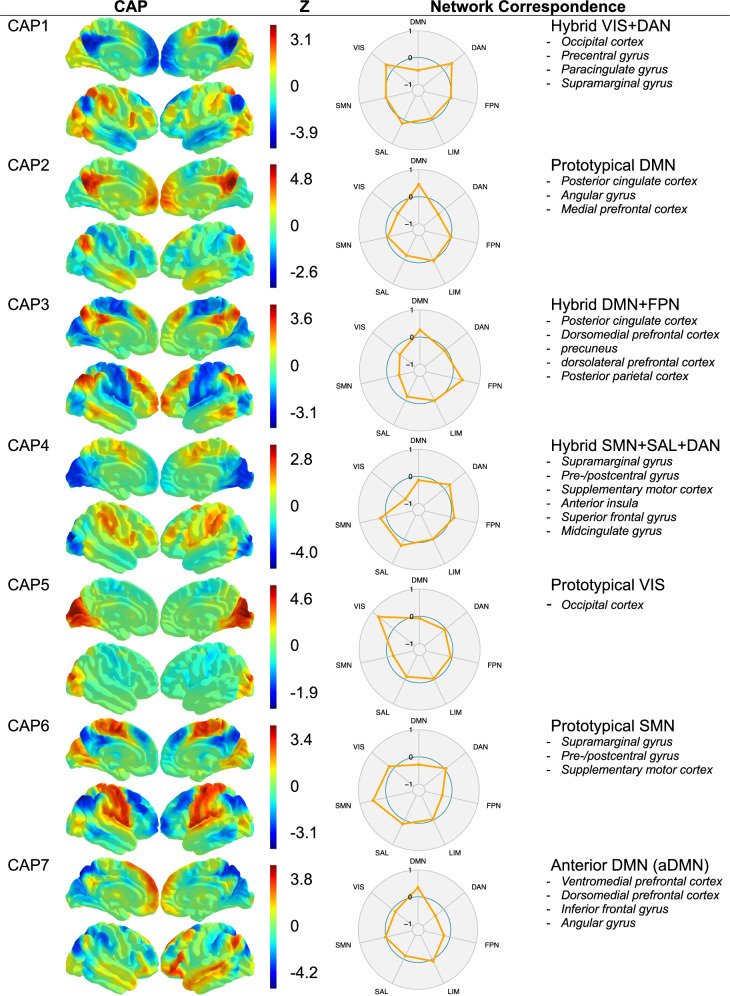
Coactivation patterns. Radar plots display the correspondence (spatial cross-correlation) between the respective CAP and the functional large-scale brain network according to the parcellation by Yeo et al. Positive values indicate activation, whereas negative values indicate deactivations in the respective network. DMN, default mode network; DAN, dorsal attention network; FPN, frontoparietal network; LIM, subcortical/limbic network; SAL, salience/ventral attention network; SMN, somatomotor network; VIS, visual network.

Consensus clustering identified 7 CAPs repeatedly present in the data. Spatial cross-correlation of z-transformed whole-brain CAP maps with Schaefer’s cortical 7 network parcellation [64] suggested CAP1 to be most strongly associated with the visual (VIS: *ρ* = 0.306) and dorsal attention network (DAN: *ρ* = 0.388). CAP2 resembled the canonical default mode network (DMN: *ρ* = 0.453). CAP3 reflected a hybrid network comprising the frontoparietal (FPN: *ρ* = 0.377) as well as the default mode network (DMN: *ρ* = 0.262), thereby representing the CAP that has been shown to relate to rumination and is of primary interest to the following analyses. CAP4 was associated with the somatomotor (SMN: *ρ* = 0.226), salience (SAL: *ρ* = 0.252) and dorsal attention networks (DAN: *ρ* = 0.250). CAP5 was linked to the canonical visual network (VIS: *ρ* = 0.715) whereas CAP6 was uniquely associated with the somatomotor network in its canonical form (SMN: *ρ* = 0.507). CAP7 primarily resembled the anterior default mode network (DMN: *ρ* = 0.346). All CAP maps as well as network correspondences are given in Fig. 2.

### Group comparisons

**Fig. 3:**
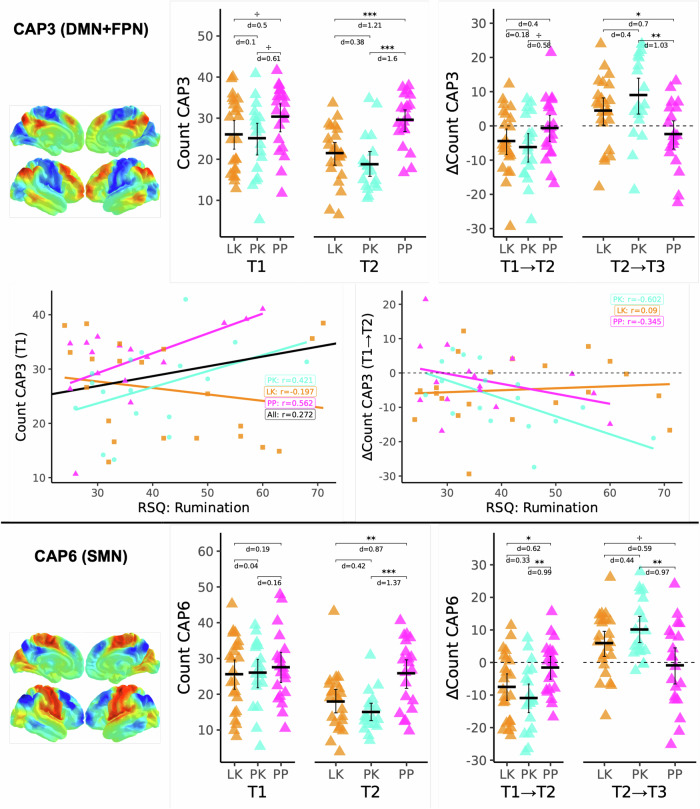
Results. At baseline (T1), self-reported rumination was positively associated with the occurrence rate of a hybrid DMN + FPN network configuration (CAP3). Ketamine acutely reduced the time that was spent in this hybrid network compared to baseline and placebo. These changes were only transient and not observable 24 h after ketamine administration. Moreover, a negative association was observed regarding rumination and ketamine induced reduction of hybrid DMN + FPN network occurrences (T1→T2). Exploratory analysis revealed that ketamine acutely reduced the time that was spent in a canonical SMN network configuration (CAP6). d, Cohens d; +, *p* < 0.1; *, *p* < 0.05; **, *p* < 0.01; ***, *p* < 0.001; LK, lamotrigine + ketamine; PK, placebo + ketamine; PP, placebo + placebo; T1, baseline; T2, during infusion; T3, 24 h after infusion. Regarding the plots on the right (within-subject changes), negative values indicate decreases, whereas positive values indicate increases in occurrence rates from Tx to Ty.

Statistics and visualizations of group differences are provided in Table 1 and Fig. 3. At baseline, CAP3 (DMN + FPN) occurrences of the PK and LK groups differed marginally compared to PP. During infusion, CAP3 occurrence differed substantially between groups, driven by strongly reduced occurrences in the PK and LK groups, compared to PP. At 24 h post infusion (T3), no meaningful differences were found between groups. Both, the PK and LK groups showed a moderate decrease of CAP3 occurrences from baseline (T1) to infusion (T2). Moreover, the decrease of CAP3 occurrence from baseline to T2 was somewhat more pronounced in the ketamine group compared to PP, effectively representing a group*time interaction. Notably, CAP3 occurrences in the PK and LK group increased back to their baseline levels from T2 to T3 and the increases were considerably greater in the PK and LK groups compared to PP.

**Table 1:** Statistics. MD, mean difference; PK, placebo + ketamine; PP, placebo + placebo; LK, lamotrigine + ketamine.

*Time point*	*MD*	*Cohens d*	*95%-CI*	*p-value*	*MD*	*Cohens d*	*95%-CI*	*p-value*	*MD*	*Cohens d*	*95%-CI*	*p-value*
	*Between-subject effects*											
CAP3	PK > PP				LK > PP				LK > PK			
T1	−5.234	−0.61	[−1.34, −0.00]	0.063	−4.306	−0.50	[−1.22, 0.07]	0.100	0.928	0.10	[−0.51, 0.72]	0.739
T2	−10.758	−1.60	[−2.84, −0.87]	< 0.001	−8.091	−1.21	[−1.96, −0.63]	< 0.001	2.667	0.38	[−0.21, 1.18]	0.224
T3	0.712	0.08	[−0.58, 0.71]	0.803	−1.145	−0.14	[−0.78, 0.47]	0.644	−1.857	−0.22	[−0.85, 0.42]	0.474
CAP6												
T1	−1.503	−0.16	[−0.75, 0.52]	0.635	−1.914	−0.19	[−0.79, 0.43]	0.535	−0.411	−0.04	[−0.67, 0.57]	0.891
T2	−10.811	−1.37	[−2.37, −0.72]	<0.001	−7.819	−0.87	[−1.71, −0.27]	0.006	2.992	0.42	[−0.17, 1.06]	0.188
T3	0.284	0.03	[−0.62, 0.65]	0.919	−0.956	−0.10	[−0.76, 0.51]	0.727	−1.239	−0.17	[−0.80, 0.46]	0.581
	*Within-subject effects*											
CAP3	PK				LK				PP			
T1 → T2	−6.299	−0.66	[−1.22, −0.26]	0.010	−4.559	−0.49	[−0.96, −0.11]	0.026	−0.774	−0.09	[−0.62, 0.35]	0.709
T2 → T3	8.895	0.73	[0.27, 1.53]	0.006	4.370	0.44	[0.04, 1.03]	0.049	−2.576	−0.27	[−0.73, 0.17]	0.244
T1 → T3	2.596	0.21	[−0.24, 0.81]	0.368	−0.189	−0.02	[−0.43, 0.43]	0.937	−3.350	−0.27	[−0.75, 0.17]	0.244
CAP6												
T1 → T2	−11.023	−1.09	[−1.84, −0.65]	<0.001	−7.620	−0.74	[−1.30, −0.35]	0.002	−1.715	−0.21	[−0.73, 0.24]	0.363
T2 → T3	10.019	1.09	[0.77, 1.59]	<0.001	5.789	0.60	[0.20, 1.22]	0.010	−1.075	−0.08	[−0.55, 0.38]	0.725
T1 → T3	−1.003	−0.10	[−0.56, 0.39]	0.663	−1.832	−0.15	[−0.65, 0.26]	0.485	−2.790	−0.23	[−0.72, 0.22]	0.306
	*Group by time interaction*											
CAP3	PK > PP				LK > PP				LK > PK			
T1 → T2	−5.534	−0.58	[−1.25, 0.03]	0.075	−3.785	−0.40	[−1.00, 0.18]	0.183	1.740	0.18	[−0.45, 0.83]	0.554
T2 → T3	11.470	1.03	[0.40, 1.84]	0.003	6.946	0.70	[0.12, 1.39]	0.026	−4.524	−0.40	[−1.10, 0.21]	0.195
T1 → T3	5.946	0.47	[−0.16, 1.17]	0.138	3.161	0.27	[−0.34, 0.91]	0.382	−2.785	−0.24	[−0.90, 0.36]	0.435
CAP6												
T1 → T2	−9.308	−0.99	[−1.75, −0.39]	0.004	−5.905	−0.62	[−1.30, −0.04]	0.045	3.402	0.33	[−0.28, 0.97]	0.279
T2 → T3	11.094	0.97	[0.39, 1.66]	0.004	6.863	0.59	[−0.01, 1.29]	0.058	−4.231	−0.44	[−1.04, 0.16]	0.164
T1 → T3	1.787	0.16	[−0.48, 0.81]	0.606	0.958	0.08	[−0.56, 0.69]	0.803	−0.828	−0.07	[−0.74, 0.49]	0.812

### Rumination

A positive association of small to moderate magnitude emerged between baseline CAP3 (DMN + FPN) occurrence and self-reported rumination *(*β = 0.272, *95%-CI* [-0.018, 0.564], *p* = 0.071, see Fig. 3). Confidence intervals indicate that the effect is likely positive and characterized by considerable variability. During infusion, the PK group exhibited a negative association of moderate magnitude between CAP3 occurrences and rumination scores, with confidence intervals indicating that the effect is likely negative and highly variable (β = -0.375, *95%-CI* [-0.985, 0.059], *p* = 0.083). For the LK and PP groups, no meaningful associations were found (LK: β = -0.105, *95%-CI* [-0.567, 0.279], *p* = 0.575; PP: β = 0.107, *95%-CI* [-0.354, 0.580], *p* = 0.622). Regarding the differences between time points, regression analysis revealed that CAP3 occurrence changes from baseline (T1) to infusion (T2) are negatively associated with rumination scores in the PK group (β = -0.602, *95%-CI* [−1.180, −0.334], *p* < 0.001). However, considerably smaller associations with inconclusive confidence intervals were found for the LK group (β = 0.090, *95%-CI* [-0.285, 0.553], *p* = 0.619) and PP group (β = -0.345, *95%-CI* [-0.815, 0.182], *p* = 0.178).

#### Exploratory analysis

After FDR-correction, groups differed significantly regarding the occurrence rate of CAP6 (SMN) during ketamine infusion (*F*(2,55) = 8.728, *p* = 0.001, *p-FDR* = 0.021). Post-hoc paired comparisons revealed substantially decreased occurrence rates of CAP6 in the PK and LK groups compared to PP. Furthermore, within-subject effects revealed decreased CAP6 occurrences from baseline to infusion, substantially exceeding the marginal decrease in the PP group, thereby indicating a treatment*time interaction. During baseline and 24 h after infusion, no group differences were observed. None of the other CAPs showed meaningful group differences after accounting for multiple testing (see table S2). Finally, all results for CAP3 and CAP6 were primarily driven by CAP persistence, as evidenced by a pronounced attenuation of all associations and group differences when persistence was included as a covariate.

## Discussion

In this randomized-controlled trial, we investigated the effects of ketamine on the occurrence rate of a previously identified dynamic correlate of rumination and whether glutamatergic modulation by lamotrigine mediated these effects. At baseline, self-reported rumination was positively associated with the occurrence rate of a hybrid DMN + FPN CAP. Ketamine acutely reduced the time that was spent in this hybrid CAP compared to baseline and placebo. These changes were only transient and not observable 24 h after ketamine administration. Moreover, an association was observed between rumination and ketamine induced reductions of hybrid DMN + FPN CAP occurrences. Exploratory analysis revealed that ketamine acutely reduced the time spent in a canonical SMN CAP. Although no substantial differences were observed between the group that was pretreated with lamotrigine compared to the mere ketamine group, effect sizes for both CAPs indicate that lamotrigine nominally diminished some of the ketamine effects.

Consistent with previous research and our hypothesis, a hybrid CAP characterized by the concurrent activation of the DMN and FPN was positively related to rumination [25, 41–43]. The simultaneous activation of the DMN and FPN is notable given their typical anticorrelation and distinct functional profiles [29, 30, 69]. The DMN is highly active at rest and considered crucial for self-referential processing [29], whereas the FPN is recruited during cognitively demanding tasks and considered central to cognitive control [30]. The positive association between DMN + FPN occurrences and rumination might therefore reflect an impaired ability to flexibly and selectively engage functionally opposing networks in individuals with higher levels of rumination. Speculatively, these impairments could contribute to difficulties disengaging from ruminative thoughts and shifting attention toward more adaptive regulatory strategies, a hallmark of rumination [70]. Notably, the effect size found here falls within the confidence interval reported by our group in a previous examination [43], supporting the robustness of this association.

During ketamine administration, occurrences of the DMN + FPN CAP previously related to rumination were strongly reduced compared to placebo. This finding is further corroborated by a more pronounced decrease from baseline to infusion in the ketamine group relative to placebo. Additionally, individuals reporting higher levels of rumination showed greater decreases in DMN + FPN CAP occurrences from baseline to ketamine administration. Notably, this association was specific to the ketamine group and did not occur in the groups receiving placebo or lamotrigine pretreatment. Given ketamine’s established effects on rumination [44], we cautiously speculate that it might exert its rapid antidepressant effects through the reduction of brain states marked by activation of functionally antagonistic networks implicated in rumination and repetitive negative thinking [25, 43]. However, because the current sample comprised healthy individuals, the translational relevance of these findings remains an open question.

Furthermore, the changes induced by ketamine were transient, indicating a fast restoration of brain homeostasis in healthy individuals. Elucidating whether the acute effects of ketamine on the DMN + FPN network configuration track its antidepressant effects might pose a promising avenue for future research. A recent study reported that a CAP primarily driven by the FPN and minor DMN contributions, occurred less frequently in treatment-resistant depression and normalized following serial ketamine infusions [45]. Consequently, our findings may not directly generalize to clinical populations. Notably, our study differs in key aspects from Taraku et al. [45]: we used voxel-wise rather than ROI-based analysis, examined effects during acute ketamine administration rather than after repeated treatments, and identified a DMN + FPN CAP with roughly equal contributions from both networks. These differences illustrate that superficially similar CAPs may not be directly comparable across studies, a common challenge in data-driven neuroimaging [71].

Exploratory analyses revealed altered dynamics of the canonical SMN following ketamine administration. During infusion, ketamine acutely reduced the time spent in the SMN compared to placebo. Additionally, the decrease in SMN occurrences from baseline to infusion was more pronounced in the ketamine group than in the placebo group. The SMN is primarily anchored in the sensorimotor and supplementary motor cortices [72], but is extensively connected with other large-scale brain networks, highlighting its role in body perception, action-related somatosensory processing, and the somatic representations of emotions [73]. Given the strong analgesic effects of ketamine [74], even at low doses, the marked reduction in time spent in the canonical SMN may reflect altered somatosensory processing during ketamine infusion. An alternative interpretation emphasizes the role of the SMN in psychomotor dysfunction across mental disorders [75]. Specifically, aberrant interactions between the SMN and other large-scale networks, such as the DMN, have been proposed to contribute to psychomotor agitation or retardation, depending on the direction of the underlying network abnormalities [75]. Furthermore, alterations within the SMN have been associated with depression [76]. In this context, our findings align with a recent report of decreased resting-state functional connectivity within a cerebellum-SMN network following repeated ketamine infusions in subjects with treatment-resistant depression [77]. Moreover, preliminary evidence indicates that perfusion changes within the SMN might emerge even after a single ketamine infusion [78]. Taken together, these findings suggest that ketamine substantially alters the temporal recruitment of circuits involved in somatosensory processing with potential implications for analgesia/anesthesia and depression. Given that the present sample consisted of healthy participants and no direct behavioral measures of somatosensory or analgesic function were acquired, the translational implications for pain processing and psychopathology remain speculative and should be interpreted with appropriate caution.

Pretreatment with lamotrigine nominally attenuated ketamine-induced changes in DMN + FPN and SMN CAP occurrences, albeit with small effect sizes and inconclusive confidence intervals. Across analyses, the lamotrigine group consistently showed smaller effect sizes than the ketamine-only group, consistent with partial glutamatergic mediation. Conversely, the marginal influence of lamotrigine might indicate that a substantial proportion of ketamine’s effects on large-scale brain dynamics is not mediated by glutamate alone. Prior task-based and resting-state fMRI studies support the role of the ketamine-induced glutamatergic surge in modulating brain circuits [11–13]. However, differences between the ketamine-only and lamotrigine-pretreated groups did not reach conventional levels of statistical significance, and these results should therefore be considered preliminary and interpreted with appropriate caution.

Several limitations of the present study should be considered. First, the sample comprised only healthy individuals, limiting the generalizability of our findings to clinical populations. Moreover, sample characteristics imply that rumination, a hallmark of depression [14, 79], might be insufficiently well represented due to the limited variability of this trait in healthy individuals. Future studies are needed to replicate these findings in clinical samples. Furthermore, the RSQ was obtained as part of an optional follow-up assessment after completion of the trial. Consequently, the timing of the RSQ assessment relative to the scanning sessions varied across participants and may have introduced additional variability into the analyses. However, because the RSQ assesses trait rumination rather than transient mood states, and because previous work has demonstrated satisfactory test–retest reliability, we believe that the measure provides a meaningful index of stable individual differences in ruminative tendencies [50]. Also, empirical evidence directly supporting lamotrigine’s inhibition of glutamatergic transmission is limited. While prior work suggests that ketamine’s effects can be attenuated by lamotrigine [9, 11–13, 80], potential differences may also reflect lamotrigine’s actions on aspartate or GABA systems [8]. Furthermore, the psychotomimetic side effects of ketamine might have resulted in functional unblinding of subjects included in the ketamine groups. The psychotomimetic side effects of ketamine themselves as well as associated changes in vigilance might also have contributed to the observed results. Another limitation pertains to the interpretation of CAPs as canonical or hybrid networks. Although CAP maps often resemble canonical resting-state networks obtained with functional connectivity approaches [40, 81], equating the two would constitute an oversimplification that may obscure minor – yet potentially informative – contributions from other networks. Other data-driven methods, like spatial ICA for example, suffer from similar drawbacks. Accordingly, we encourage a cautious and critical interpretation of CAP-to-network assignments. At the same time, some degree of spatial simplification is necessary to facilitate comparison across studies and to support interpretability. Finally, the relatively small sample size underscores the need for replication in larger cohorts.

## Conclusion

In this randomized, double-blind, placebo-controlled fMRI study, ketamine acutely reduced the occurrence of a hybrid DMN + FPN coactivation pattern. The DMN + FPN configuration was linked to self-reported rumination, and stronger decreases were observed in individuals reporting higher rumination. These changes were transient and parallelled by reduced time spent in a canonical somatomotor network. Pretreatment with lamotrigine nominally attenuated ketamine’s effects across analyses, consistent with partial glutamatergic mediation. Together, our findings provide evidence that ketamine modulates large-scale brain network dynamics associated with rumination and somatosensory processing in healthy individuals, offering mechanistic insights into its rapid antidepressant and antiruminative effects.

## Supplementary information

Supplemental Material

## Data Availability

Code and data used in this article can be provided upon reasonable request. Enquiries can be directed to the corresponding author.

## References

[1] McIntyre RS, Carvalho IP, Lui LMW, Majeed A, Masand PS, Gill H, et al. The effect of intravenous, intranasal, and oral ketamine in mood disorders: a meta-analysis. J Affect Disord. 2020;276:576–84. 32871689 10.1016/j.jad.2020.06.050

[2] Meiering MS, Weigner D, Gärtner M, Schäfer T, Grimm S. Does route of administration affect antidepressant efficacy of ketamine? A meta-analysis of double-blind randomized controlled trials comparing intravenous and intranasal administration. J Psychiatr Res. 2022;156:639–46. 36375231 10.1016/j.jpsychires.2022.10.062

[3] Whittaker E, Dadabayev AR, Joshi SA, Glue P. Systematic review and meta-analysis of randomized controlled trials of ketamine in the treatment of refractory anxiety spectrum disorders. Therapeutic Adv Psychopharmacol. 2021;11:204512532110567. 10.1177/20451253211056743 PMC8679040 34925757

[4] Abdallah CG, Adams TG, Kelmendi B, Esterlis I, Sanacora G, Krystal JH. Ketamine’s mechanism of action: a path to rapid-acting antidepressants. Depress Anxiety. 2016;33:689–97. 27062302 10.1002/da.22501 PMC4961540

[5] Abdallah CG, Sanacora G, Duman RS, Krystal JH. The neurobiology of depression, ketamine and rapid-acting antidepressants: is it glutamate inhibition or activation?. Pharmacology Therapeutics. 2018;190:148–58. 29803629 10.1016/j.pharmthera.2018.05.010 PMC6165688

[6] Lullau APM, Haga EMW, Ronold EH, Dwyer GE. Antidepressant mechanisms of ketamine: a review of actions with relevance to treatment-resistance and neuroprogression. Front Neurosci. 2023;17:1223145. 37614344 10.3389/fnins.2023.1223145 PMC10442706

[7] Liao C, Dua AN, Wojtasiewicz C, Liston C, Kwan AC. Structural neural plasticity evoked by rapid-acting antidepressant interventions. Nat Rev Neurosci. 2025;26:101–14. 39558048 10.1038/s41583-024-00876-0 PMC11892022

[8] Leach MJ, Marden CM, Miller AA. Pharmacological studies on lamotrigine, a novel potential antiepileptic drug: II. neurochemical studies on the mechanism of action. Epilepsia. 1986;27:490–7. 3757936 10.1111/j.1528-1157.1986.tb03573.x

[9] Doyle OM, De Simoni S, Schwarz AJ, Brittain C, O’Daly OG, Williams SCR, et al. Quantifying the attenuation of the ketamine pharmacological magnetic resonance imaging response in humans: a validation using antipsychotic and glutamatergic agents. J Pharmacol Exp Ther. 2013;345:151–60. 23370794 10.1124/jpet.112.201665

[10] Gärtner M, Weigand A, Keicher C, Meiering MS, Weigner D, Carstens L, et al. Modulatory effects of ketamine and lamotrigine on cognition: emotion interaction in the brain. Neuropsychobiology. 2023;82:91–103. 36731434 10.1159/000528315

[11] Gärtner M, Weigand A, Meiering MS, Weigner D, Carstens L, Keicher C, et al. Region- and time- specific effects of ketamine on cerebral blood flow: a randomized controlled trial. Neuropsychopharmacol. 2023;48:1735–41. 10.1038/s41386-023-01605-4 PMC10579356 37231079

[12] Meiering MS, Weigner D, Gärtner M, Carstens L, Keicher C, Hertrampf R, et al. Functional activity and connectivity signatures of ketamine and lamotrigine during negative emotional processing: a double-blind randomized controlled fMRI study. Transl Psychiatry. 2024;14:436. 39402015 10.1038/s41398-024-03120-6 PMC11479267

[13] Weigner D, Meiering MS, Weigand A, Carstens L, Keicher C, Hertrampf R, et al. Functional connectivity alterations of the pregenual anterior cingulate cortex by ketamine and the modulation by lamotrigine. J Psychopharmacol. 2025;39:1154–63. 40536009 10.1177/02698811251346705

[14] Ehring T, Watkins ER. Repetitive negative thinking as a transdiagnostic process. Int J Cognit Ther. 2008;1:192–205.

[15] Moulds ML, McEvoy PM. Repetitive negative thinking as a transdiagnostic cognitive process. Nat Rev Psychol. 2025;4:127–41.

[16] Nolen-Hoeksema S, Wisco BE, Lyubomirsky S. Rethinking rumination. Perspect Psychol Sci. 2008;3:400–24. 26158958 10.1111/j.1745-6924.2008.00088.x

[17] Nolen-Hoeksema S, Morrow J. A prospective study of depression and posttraumatic stress symptoms after a natural disaster: the 1989 loma prieta earthquake. J Personality Soc Psychol. 1991;61:115–21. 10.1037//0022-3514.61.1.115 1890582

[18] Nolen-Hoeksema S, Watkins ER. A heuristic for developing transdiagnostic models of psychopathology: explaining multifinality and divergent trajectories. Perspect Psychol Sci. 2011;6:589–609. 26168379 10.1177/1745691611419672

[19] Arditte KA, Shaw AM, Timpano KR. Repetitive negative thinking: a transdiagnostic correlate of affective disorders. J Soc Clin Psychol. 2016;35:181–201.

[20] Colpizzi I, Pössel P, Marchetti I. Unveiling the necessary conditions for depressive symptoms in adolescent girls and boys: a necessary condition analysis study. J Youth Adolescence. 2025;54:2494–507. 10.1007/s10964-025-02220-w 40637931

[21] Marchetti I, Koster EHW, Hankin BL. Which psychosocial risks are necessary for developing depression during adolescence? A novel approach applying necessary condition analysis. J Am Acad Child Adolesc Psychiatry. 2025;64:1201–9. 39522678 10.1016/j.jaac.2024.11.001

[22] Struijs SY, De Jong PJ, Jeronimus BF, Van Der Does W, Riese H, Spinhoven P. Psychological risk factors and the course of depression and anxiety disorders: a review of 15 years NESDA research. J Affect Disord. 2021;295:1347–59. 34706448 10.1016/j.jad.2021.08.086

[23] Hamilton JP, Furman DJ, Chang C, Thomason ME, Dennis E, Gotlib IH. Default-mode and task-positive network activity in major depressive disorder: implications for adaptive and maladaptive rumination. Biol Psychiatry. 2011;70:327–33. 21459364 10.1016/j.biopsych.2011.02.003 PMC3144981

[24] Lydon-Staley DM, Kuehner C, Zamoscik V, Huffziger S, Kirsch P, Bassett DS. Repetitive negative thinking in daily life and functional connectivity among default mode, fronto-parietal, and salience networks. Transl Psychiatry. 2019;9:234. 31534117 10.1038/s41398-019-0560-0 PMC6751201

[25] Peterson EC, Smolker HR, Moser AD, Kaiser RH. Review of dynamic resting-state methods in neuroimaging: applications to depression and rumination. Hum Brain Mapp. 2025;46:e70377. 41104784 10.1002/hbm.70377 PMC12532080

[26] Singh S, Shaw SB, Becker S. Reduced task-switching flexibility in parietal-cingulate and frontal circuits associated with brooding. Cogn Neurodyn. 2025;20:55 10.31234/osf.io/yczs9_v1. 10.1007/s11571-026-10425-3 PMC12920956 41728210

[27] Zhou D, Ahn J, Lydon-Staley DM, Falk EB, Bessett DS, Ruscio AM Neural dynamics underlying the persistence of perseverative thought in depression and anxiety. Biorxiv [Preprint]. 2025. Available from: https://www.biorxiv.org/content/10.1101/2025.07.21.666019v1.

[28] Menon V. Large-scale brain networks and psychopathology: a unifying triple network model. Trends Cognit Sci. 2011;15:483–506. 21908230 10.1016/j.tics.2011.08.003

[29] Menon V. 20 years of the default mode network: a review and synthesis. Neuron. 2023;111:2469–87. 37167968 10.1016/j.neuron.2023.04.023 PMC10524518

[30] Menon V, D’Esposito M. The role of PFC networks in cognitive control and executive function. Neuropsychopharmacol. 2022;47:90–103. 10.1038/s41386-021-01152-w PMC8616903 34408276

[31] Seeley WW. The salience network: a neural system for perceiving and responding to homeostatic demands. J Neurosci. 2019;39:9878–82. 31676604 10.1523/JNEUROSCI.1138-17.2019 PMC6978945

[32] Seeley WW, Menon V, Schatzberg AF, Keller J, Glover GH, Kenna H, et al. Dissociable intrinsic connectivity networks for salience processing and executive control. J Neurosci. 2007;27:2349–56. 17329432 10.1523/JNEUROSCI.5587-06.2007 PMC2680293

[33] Sridharan D, Levitin DJ, Menon V. A critical role for the right fronto-insular cortex in switching between central-executive and default-mode networks. Proc Natl Acad Sci USA. 2008;105:12569–74. 18723676 10.1073/pnas.0800005105 PMC2527952

[34] Uddin LQ. Salience processing and insular cortical function and dysfunction. Nat Rev Neurosci. 2015;16:55–61. 25406711 10.1038/nrn3857

[35] Makovac E, Fagioli S, Rae CL, Critchley HD, Ottaviani C. Can’t get it off my brain: Meta-analysis of neuroimaging studies on perseverative cognition. Psychiatry Research: Neuroimaging. 2020;295:111020. 31790922 10.1016/j.pscychresns.2019.111020

[36] Allen EA, Damaraju E, Plis SM, Erhardt EB, Eichele T, Calhoun VD. Tracking whole-brain connectivity dynamics in the resting state. Cereb Cortex. 2014;24:663–76. 23146964 10.1093/cercor/bhs352 PMC3920766

[37] Breakspear M. Dynamic models of large-scale brain activity. Nat Neurosci. 2017;20:340–52. 28230845 10.1038/nn.4497

[38] Chen JE, Chang C, Greicius MD, Glover GH. Introducing co-activation pattern metrics to quantify spontaneous brain network dynamics. NeuroImage. 2015;111:476–88. 25662866 10.1016/j.neuroimage.2015.01.057 PMC4386757

[39] Bolton TAW, Tuleasca C, Wotruba D, Rey G, Dhanis H, Gauthier B, et al. TbCAPs: a toolbox for co-activation pattern analysis. NeuroImage. 2020;211:116621. 32058000 10.1016/j.neuroimage.2020.116621

[40] Liu X, Chang C, Duyn JH. Decomposition of spontaneous brain activity into distinct fMRI co-activation patterns. Front Syst Neurosci. 2013;7:1–11. 24550788 10.3389/fnsys.2013.00101 PMC3913885

[41] Belleau EL, Bolton TAW, Kaiser RH, Clegg R, Cárdenas E, Goer F, et al. Resting state brain dynamics: associations with childhood sexual abuse and major depressive disorder. NeuroImage: Clin. 2022;36:103164. 36044792 10.1016/j.nicl.2022.103164 PMC9449675

[42] Kaiser RH, Kang MS, Lew Y, Van Der Feen J, Aguirre B, Clegg R, et al. Abnormal frontoinsular-default network dynamics in adolescent depression and rumination: a preliminary resting-state co-activation pattern analysis. Neuropsychopharmacol. 2019;44:1604–12. 10.1038/s41386-019-0399-3 PMC6784913 31035283

[43] Meiering MS, Belleau E, Weigner D, Gruzman R, Pizzagalli D, Enge S, et al. Dynamic coactivation patterns during repetitive negative thinking: a cross-sectional fMRI study. Psychol Med. 2026;56:1–12. 10.1017/S0033291726103572 PMC12969223 41782393

[44] Vidal S, Jermann F, Aubry J-M, Richard-Lepouriel H, Kosel M. Effect of ketamine on rumination in treatment-resistant depressive patients. J Clin Psychopharmacol. 2020;40:607–10. 33044358 10.1097/JCP.0000000000001305

[45] Taraku B, Nomi JS, Zavaliangos-Petropulu A, Al-Sharif N, Pfeiffer P, Norris V, et al. Modulation of functional network co-activation pattern dynamics following ketamine treatment in major depression. Imaging Neurosci. 2025;3:IMAG.a.936. 10.1162/IMAG.a.936 PMC12529346 41113939

[46] Faul F, Erdfelder E, Lang A-G, Buchner A. G*Power 3: a flexible statistical power analysis program for the social, behavioral, and biomedical sciences. Behav Res Methods. 2007;39:175–91. 17695343 10.3758/bf03193146

[47] Cohen AF, Land GS, Breimer DD, Yuen WC, Winton C, Peck AW. Lamotrigine, a new anticonvulsant: pharmacokinetics in normal humans. Clin Pharmacol Ther. 1987;42:535–41. 3677542 10.1038/clpt.1987.193

[48] Gärtner M, Weigand A, Meiering MS, Weigner D, Carstens L, Keicher C, et al. Negative emotionality shapes the modulatory effects of ketamine and lamotrigine in subregions of the anterior cingulate cortex. Transl Psychiatry. 2024;14:2–7. 38890270 10.1038/s41398-024-02977-x PMC11189565

[49] Bürger C, Kühner C. Copingstile im umgang mit depressiver stimmung. Z Klin Psychol Psychother. 2007;36:36–45.

[50] Kühner C, Huffziger S, Nolen-Hoeksema S RSQ-D: Response Styles Questionaire -Deutsche Version-. Hogrefe: Göttingen, 2007.

[51] Woolrich MW, Behrens TEJ, Beckmann CF, Jenkinson M, Smith SM. Multilevel linear modelling for FMRI group analysis using Bayesian inference. NeuroImage. 2004;21:1732–47. 15050594 10.1016/j.neuroimage.2003.12.023

[52] Woolrich MW, Behrens TEJ, Smith SM. Constrained linear basis sets for HRF modelling using Variational Bayes. NeuroImage. 2004;21:1748–61. 15050595 10.1016/j.neuroimage.2003.12.024

[53] Woolrich MW, Ripley BD, Brady M, Smith SM. Temporal autocorrelation in univariate linear modeling of FMRI data. NeuroImage. 2001;14:1370–86. 11707093 10.1006/nimg.2001.0931

[54] Jenkinson M, Beckmann CF, Behrens TEJ, Woolrich MW, Smith SM. FSL. NeuroImage. 2012;62:782–90. 21979382 10.1016/j.neuroimage.2011.09.015

[55] Smith SM, Jenkinson M, Woolrich MW, Beckmann CF, Behrens TEJ, Johansen-Berg H, et al. Advances in functional and structural MR image analysis and implementation as FSL. NeuroImage. 2004;23:208–19. 10.1016/j.neuroimage.2004.07.051 15501092

[56] Woolrich MW, Jbabdi S, Patenaude B, Chappell M, Makni S, Behrens T, et al. Bayesian analysis of neuroimaging data in FSL. NeuroImage. 2009;45:173–86. 10.1016/j.neuroimage.2008.10.055 19059349

[57] Pruim RHR, Mennes M, Van Rooij D, Llera A, Buitelaar JK, Beckmann CF. ICA-AROMA: a robust ICA-based strategy for removing motion artifacts from fMRI data. NeuroImage. 2015;112:267–77. 25770991 10.1016/j.neuroimage.2015.02.064

[58] Pruim RHR, Mennes M, Buitelaar JK, Beckmann CF. Evaluation of ICA-AROMA and alternative strategies for motion artifact removal in resting state fMRI. NeuroImage. 2015;112:278–87. 25770990 10.1016/j.neuroimage.2015.02.063

[59] Friston KJ, Williams S, Howard R, Frackowiak RSJ, Turner R. Movement-Related effects in fMRI time-series. Magnetic Reson Med. 1996;35:346–55. 10.1002/mrm.1910350312 8699946

[60] Iraji A, Faghiri A, Fu Z, Kochunov P, Adhikari BM, Belger A, et al. Moving beyond the ‘CAP’ of the Iceberg: Intrinsic connectivity networks in fMRI are continuously engaging and overlapping. NeuroImage. 2022;251:119013. 35189361 10.1016/j.neuroimage.2022.119013 PMC9107614

[61] Monti S, Tamayo P, Mesirov J, Golub T. Consensus clustering: a resampling-based method for class discovery and visualization of gene expression microarray data. Mach Learn. 2003;52:91–118.

[62] Zoller DM, Bolton TAW, Karahanoglu FI, Eliez S, Schaer M, Van De Ville D. Robust recovery of temporal overlap between network activity using transient-informed spatio-temporal regression. IEEE Trans Med Imaging. 2019;38:291–302. 30188815 10.1109/TMI.2018.2863944

[63] Șenbabaoğlu Y, Michailidis G, Li JZ. Critical limitations of consensus clustering in class discovery. Sci Rep. 2014;4:6207. 25158761 10.1038/srep06207 PMC4145288

[64] Schaefer A, Kong R, Gordon EM, Laumann TO, Zuo X-N, Holmes AJ, et al. Local-global parcellation of the human cerebral cortex from intrinsic functional connectivity MRI. Cereb Cortex. 2018;28:3095–114. 28981612 10.1093/cercor/bhx179 PMC6095216

[65] R Core Team. R: A language and environment for statistical computing. 2023. https://www.r-project.org/. Accessed 16 May2023.

[66] Cumming G. The new statistics: why and how. Psychol Sci. 2014;25:7–29. 24220629 10.1177/0956797613504966

[67] Cumming G, Finch S. Inference by eye: confidence intervals and how to read pictures of data. Am Psychologist. 2005;60:170–80. 10.1037/0003-066X.60.2.170 15740449

[68] Frossard J, Renaud O. Permutation tests for regression, ANOVA, and comparison of signals: the permuco package. J Stat Soft. 2021;99:1–32. 10.18637/jss.v099.i15.

[69] Fox MD, Snyder AZ, Vincent JL, Corbetta M, Van Essen DC, Raichle ME. The human brain is intrinsically organized into dynamic, anticorrelated functional networks. Proc Natl Acad Sci USA. 2005;102:9673–8. 15976020 10.1073/pnas.0504136102 PMC1157105

[70] Koster EHW, De Lissnyder E, Derakshan N, De Raedt R. Understanding depressive rumination from a cognitive science perspective: the impaired disengagement hypothesis. Clin Psychol Rev. 2011;31:138–45. 20817334 10.1016/j.cpr.2010.08.005

[71] Bijsterbosch J, Smith SM, Beckmann C Introduction to resting state fMRI functional connectivity. First edition. Oxford University Press: Oxford, United Kingdom, 2017.

[72] Habas C, Kamdar N, Nguyen D, Prater K, Beckmann CF, Menon V, et al. Distinct cerebellar contributions to intrinsic connectivity networks. J Neurosci. 2009;29:8586–94. 19571149 10.1523/JNEUROSCI.1868-09.2009 PMC2742620

[73] De Haan EHF, Dijkerman HC. Somatosensation in the brain: a theoretical Re-evaluation and a new model. Trends Cognit Sci. 2020;24:529–41. 32430229 10.1016/j.tics.2020.04.003

[74] Ma X, Yan J, Jiang H. Application of ketamine in pain management and the underlying mechanism. Pain Res Manag. 2023;2023:1–11. 10.1155/2023/1928969 PMC10447145 37622028

[75] Northoff G, Hirjak D, Wolf RC, Magioncalda P, Martino M. All roads lead to the motor cortex: psychomotor mechanisms and their biochemical modulation in psychiatric disorders. Mol Psychiatry. 2021;26:92–102. 32555423 10.1038/s41380-020-0814-5

[76] Zhang Z, Zhang Y, Wang H, Lei M, Jiang Y, Xiong D, et al. Resting-state network alterations in depression: a comprehensive meta-analysis of functional connectivity. Psychol Med. 2025;55:e63. 40008424 10.1017/S0033291725000303 PMC12080655

[77] Sahib AK, Loureiro JR, Vasavada M, Anderson C, Kubicki A, Wade B, et al. Modulation of the functional connectome in major depressive disorder by ketamine therapy. Psychol Med. 2022;52:2596–605. 33267926 10.1017/S0033291720004560 PMC9647551

[78] Sahib AK, Loureiro JRA, Vasavada MM, Kubicki A, Joshi SH, Wang K, et al. Single and repeated ketamine treatment induces perfusion changes in sensory and limbic networks in major depressive disorder. Eur Neuropsychopharmacol. 2020;33:89–100. 32061453 10.1016/j.euroneuro.2020.01.017 PMC8869841

[79] Ehring T. Thinking too much: rumination and psychopathology. World Psychiatry. 2021;20:441–2. 34505392 10.1002/wps.20910 PMC8429319

[80] Deakin JFW, Lees J, McKie S, Hallak JEC, Williams SR, Dursun SM. Glutamate and the neural basis of the subjective effects of ketamine: a pharmaco–magnetic resonance imaging study. Arch Gen Psychiatry. 2008;65:154–64. 18250253 10.1001/archgenpsychiatry.2007.37

[81] Bolton TAW TbCAPs User Manual. 2020. https://github.com/FabienCarruzzo/tbCAPS.

