# Supplementary material for: Ketamine’s impact on rumination-related brain dynamics: insights from a randomized controlled fMRI trial
Source: Transl Psychiatry. 2026 Aug 18;16:417. doi: 10.1038/s41398-026-04392-w (PMC13487162; doi:10.1038/s41398-026-04392-w)
Supplement: Supplementary file 1 [file 41398_2026_4392_MOESM1_ESM.pdf]

Supplement

to

Ketamine's Impact on Rumination-Related Brain Dynamics: Insights From a Randomized Controlled fMRI Trial

Marvin S. Meiering MSc[1,2], David Weigner MSc[1,2], Matti Gärtner PhD[1], Luisa Carstens PhD[1], Christian Keicher MD[3], Gerd Luippold MD[4], Andreas Wunder PhD[5], Anne Weigand PhD[1], & Simone Grimm PhD[1,6]

[1]Institute of Neuroscience and Biopsychology for Clinical Application, Medical School Berlin, Berlin, Germany

[2]Department of Psychology, Freie Universität Berlin, Berlin, Germany

[3]Charité Research Organisation GmbH, Berlin, Germany

[4]Experimental Medicine, Boehringer Ingelheim Pharma GmbH & Co. KG, Biberach an Der Riss, Germany.

[5]Free Consultant, Lübeck, Germany

[6]Department of Psychiatry, Psychotherapy and Psychosomatics, University Hospital of Psychiatry, University of Zurich, Zurich, Switzerland

Corresponding Author:

Marvin S. Meiering (marvin.meiering@medicalschool-berlin.de)

Institute of Neuroscience and Biopsychology for Clinical Application

MSB Medical School Berlin

Rüdesheimer Straße 50

D-14197 Berlin

Figure S1. Flow chart

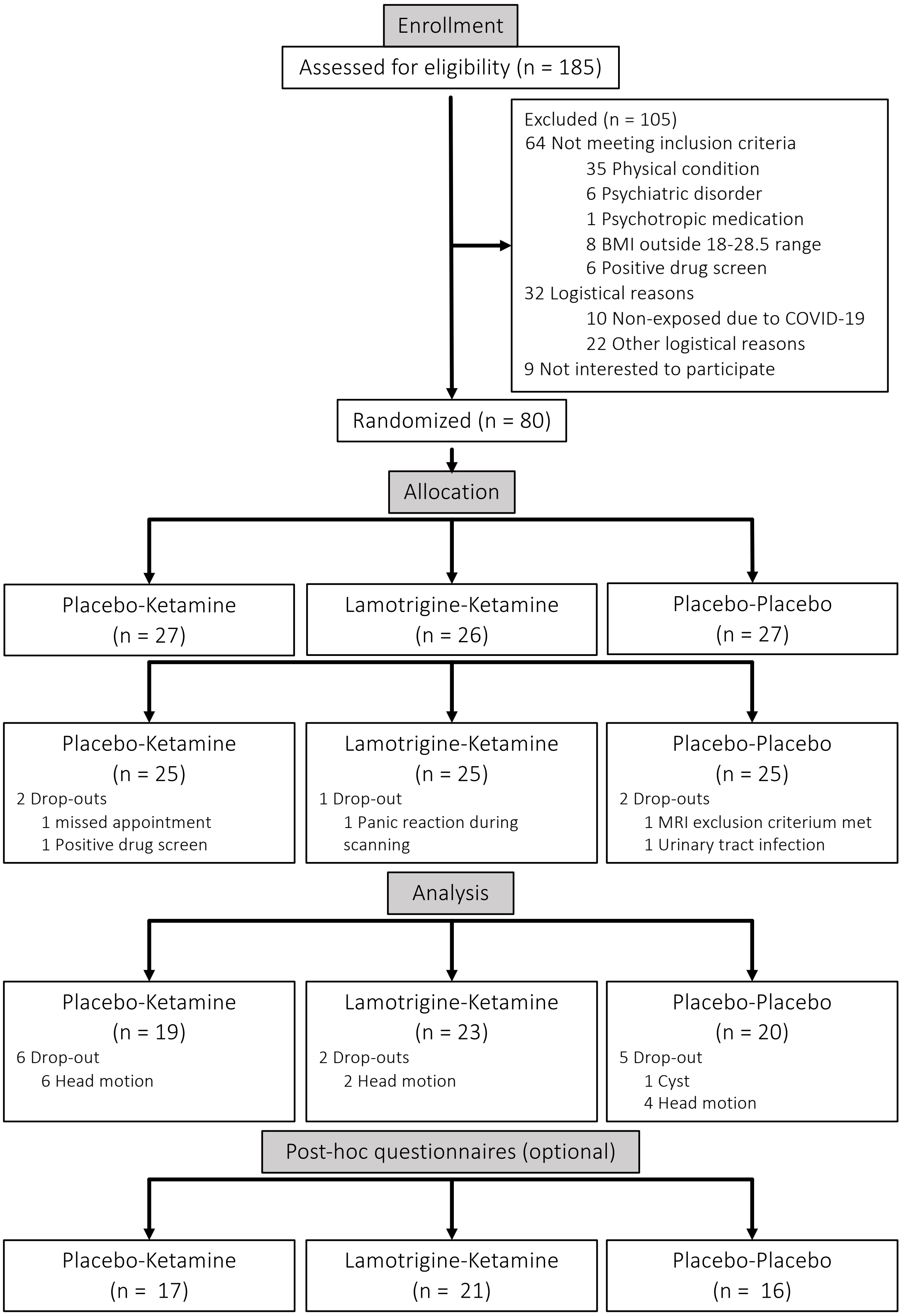

MRI image acquisition and analysis

MRI Acquisition. Brain images were acquired using a 3 Tesla MRI scanner (PRISMA fit, Siemens Medical Systems, Erlangen, Germany). The anatomical image was acquired by means of a 3D T1 weighted sequence (Magnetization Prepared Rapid Acquisition Gradient Echo sequence, TR = 2.3 s, TE = 3.03 ms, slices = 192, voxel size = 1x1x1 mm, flip angle = 9°, FOV = 256x256x192 mm). Functional brain images were acquired using a T2* weighted gradient echo-planar imaging sequence sensitive to the BOLD effect (TR = 2 s, TE = 30 ms, flip angle = 80°, voxel size = 3x3x3 mm, matrix 64x64, 36 slices, FOV = 192x192x143 mm, GRAPPA acceleration factor 2). Moreover, to improve registration a fieldmap was obtained employing a double-echo gradient echo field map sequence (TR = 468 ms, TE = 4.92 / 7.38 ms, slices = 39, voxel size = 3x3x3 mm, flip angle = 60°, matrix = 64x64, FOV = 192x192x140 mm).

Preprocessing. Brain image preprocessing and analysis were carried out using FEAT (FMRI Expert Analysis Tool; (Woolrich, Behrens, Beckmann, et al., 2004; Woolrich, Behrens, & Smith, 2004; Woolrich et al., 2001)) version 6, as part of FSL (FMRIB's Software Library; (Jenkinson et al., 2012; Smith et al., 2004; Woolrich et al., 2009)). T1 anatomical data were biasfield corrected and aligned to the MNI152 standard space using linear alignment via FSL FLIRT with 12 degrees of freedom and subsequently refined non-linearly as implemented in FSL FNIRT. Furthermore, a transformation from the functional space to the T1 anatomical space using FSL Boundary Based Registration was obtained. Eventually, the transformation was combined with the T1 to MNI152 registration to transfer the functional data from the individual's native space to the MNI152 standard space. The processing of the functional brain images included correction for participant head motion, correction for EPI distortions using fieldmap data, and a 5 mm FWHM spatial smoothing. To identify and correct for more subtle effects of head motion FSL's MELODIC (Beckmann & Smith, 2004, 2005) was used to extract independent data components followed by ICA-AROMA (Pruim, Mennes, Buitelaar, et al., 2015; Pruim, Mennes, Van Rooij, et al., 2015) to identify and remove secondary effects of head motion. Next, BOLD signal from the cerebrospinal fluid and white matter together with 6 motion parameters, their derivatives and polynomials were regressed from the time series. Finally, spike regression and global signal regression were performed to remove any residual effects of head motion, and a temporal 0.01 Hz high-pass filter was applied to account for scanner drifts.

Figure S2. Clustering accuracy for k=5 to k=11

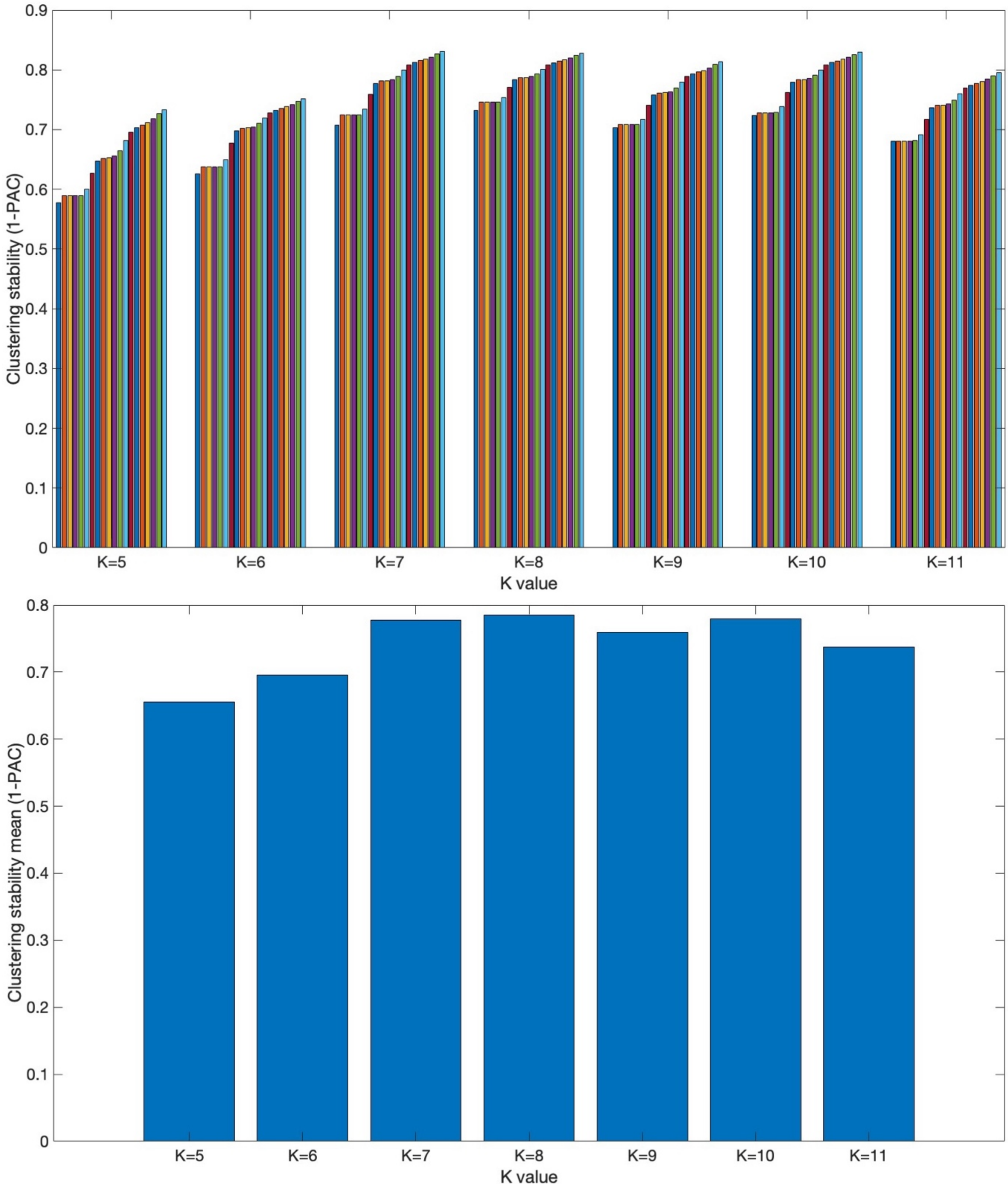

Notes. Higher values reflect better clustering accuracy. PAC, proportion of ambiguously clustered pairs. The color gradient for a given cluster number denotes the assessment of clustering quality with a more or less strict definition of "ambiguous clustering" (strictness decreases from left to right).

Figure S3. Delta fitting for clustering stability for k=5 to k=11.

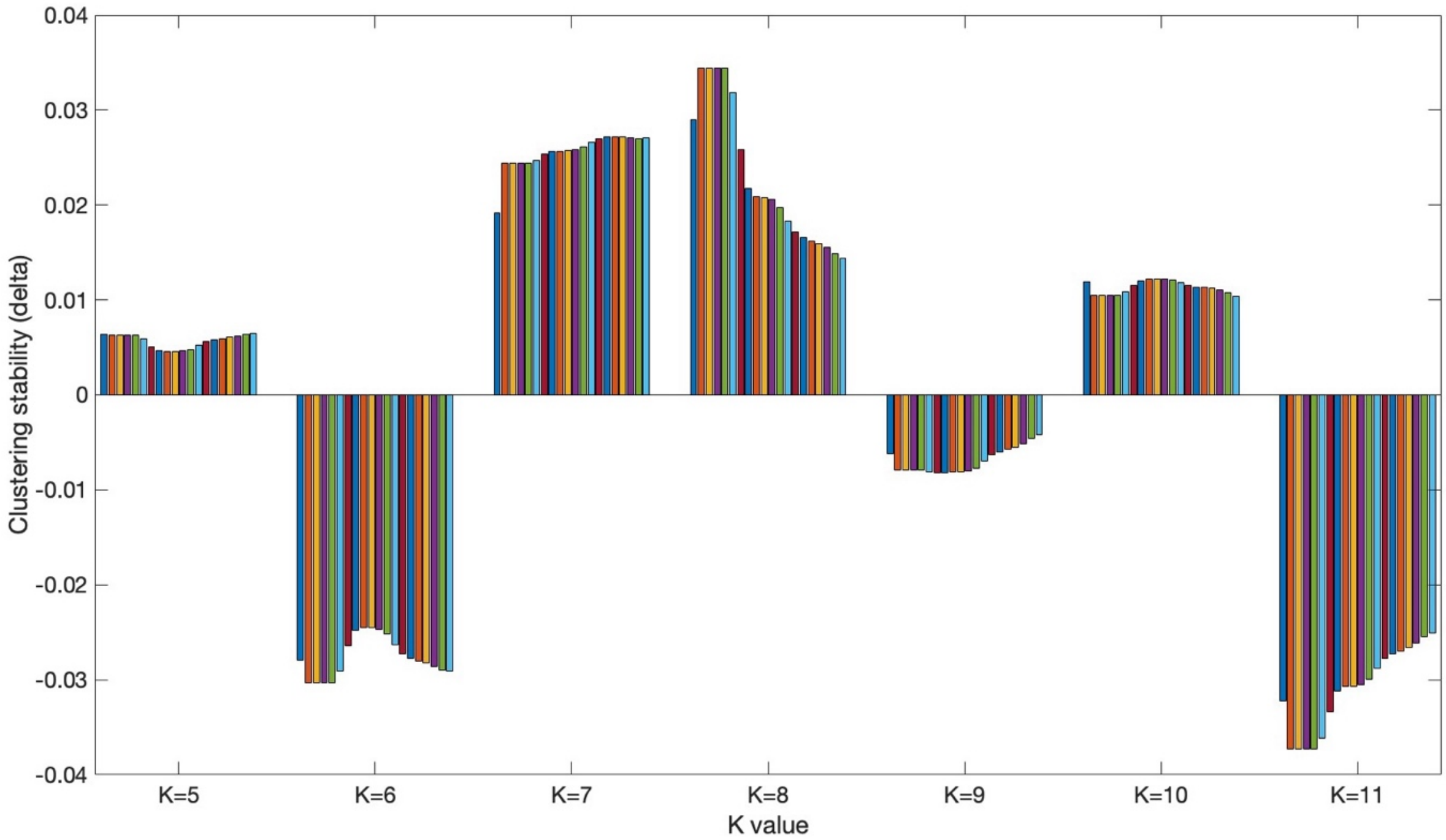

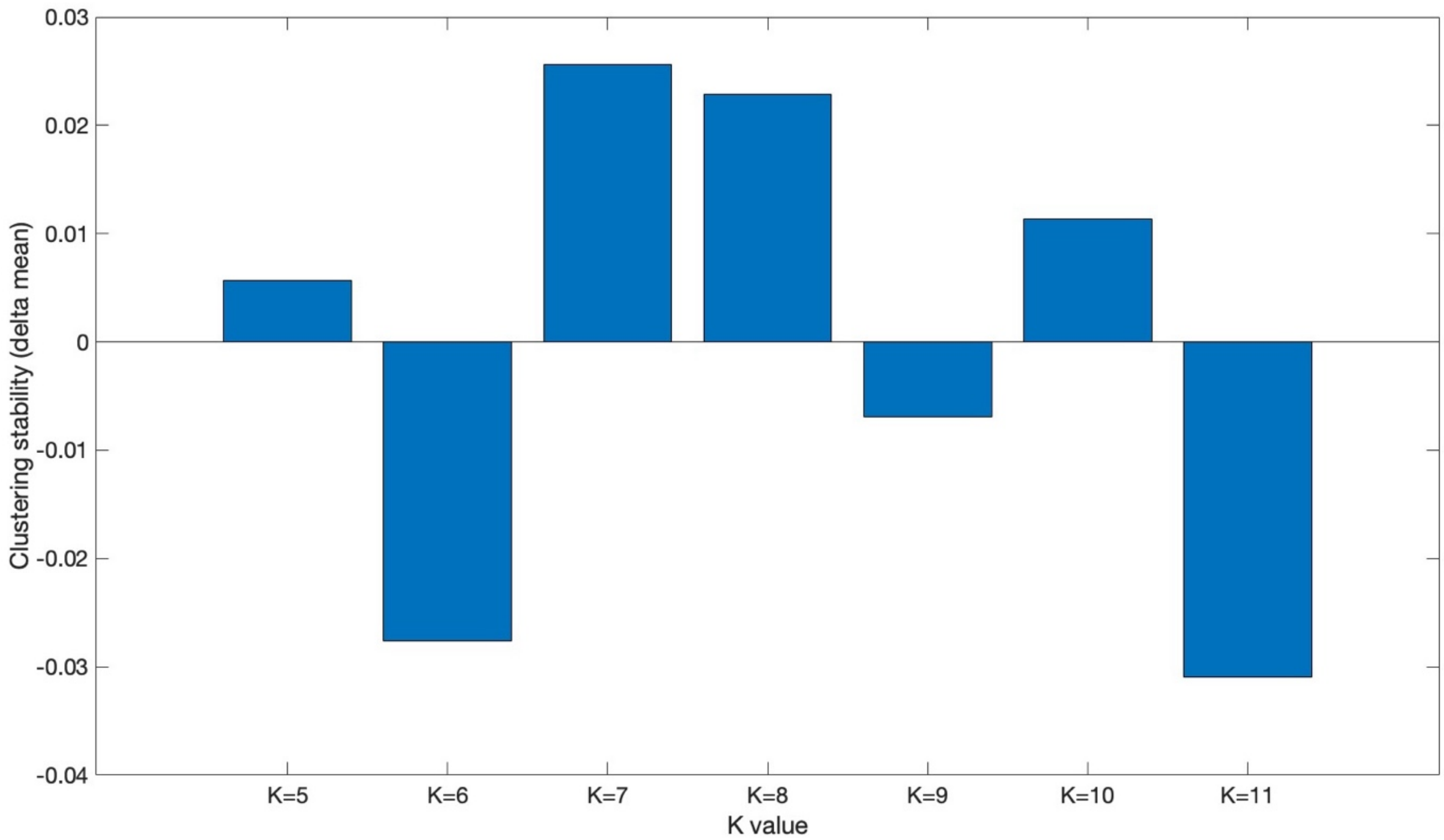

Notes. Here, the PAC values are fitted against a delta function. Accordingly, higher values reflect better clustering accuracy, after accounting for the stability increase that is expected as a function of k. The clustering solution k = 7 was determined as the winning parameter, since it performs considerably better than would be expected based solely on increases of k. The clustering solution with k=8 also performed similarly well compared to k=7. However, visual inspection of the clustering quality across different definitions of stability indicated substantial variability of the clustering quality. PAC, proportion of ambiguously clustered pairs. The color gradient for a given cluster number denotes the assessment of clustering quality with a more or less strict definition of "ambiguous clustering" (strictness decreases from left to right).

Table S1. Spatial cross-correlations between CAPs and Yeo's 7 network atlas.

CAP	DMN	DAN	FPN	LIM	VAN/SAL	SMN	VN
1	-0.477	0.388	0.034	-0.055	0.151	0.012	0.306
2	0.453	-0.301	0.001	0.056	-0.166	-0.037	-0.237
3	0.262	-0.064	0.377	0.010	-0.150	-0.437	-0.278
4	-0.138	0.250	0.128	-0.008	0.252	0.226	-0.601
5	-0.076	-0.045	-0.050	-0.035	-0.116	-0.228	0.715
6	-0.284	0.051	-0.339	-0.032	0.161	0.507	0.174
7	0.346	-0.390	-0.227	0.081	-0.139	0.022	-0.140

Notes. CAP, coactivation pattern; DMN, default mode network; DAN, dorsal attention network; FPN, frontoparietal network; LIM, limbic network; VAN, ventral attention network; SMN, somatomotor network; VN, visual network. Positive values indicate an overlap of the respective network with activations within the CAP map. Negative values indicate an overlap of the respective network with deactivations within the CAP map.

Table S2. ANCOVA: Analyses of group differences

CAP	T1	T2	T3	T1→T2	T2→T3	T1→T3
Uncorrected p-values						
CAP1	.441	.096	.743	.589	.294	.825
CAP2	.617	.063	.949	.581	.110	.718
CAP3	.158	**<.0001**	.801	.171	**.005**	.298
CAP4	.599	.135	.159	.064	.036	.203
CAP5	.801	.210	.768	.173	.195	.571
CAP6	.824	**.001**	.900	**.014**	**.010**	.894
CAP7	.650	.145	.999	.646	.288	.699
FDR-corrected p-values						
CAP1	.805	.448	.912	.910	.569	.912
CAP2	.910	.336	.972	.910	.454	.912
CAP3	.454	**.004**	.912	.454	.070	.569
CAP4	.910	.454	.454	.336	.252	.464
CAP5	.912	.464	.912	.454	.464	.910
CAP6	.912	**.021**	.945	.118	.105	.945
CAP7	.910	.454	.999	.910	.569	.912

Notes. Significant p-values are highlighted in bold.

Figure S4. Group differences

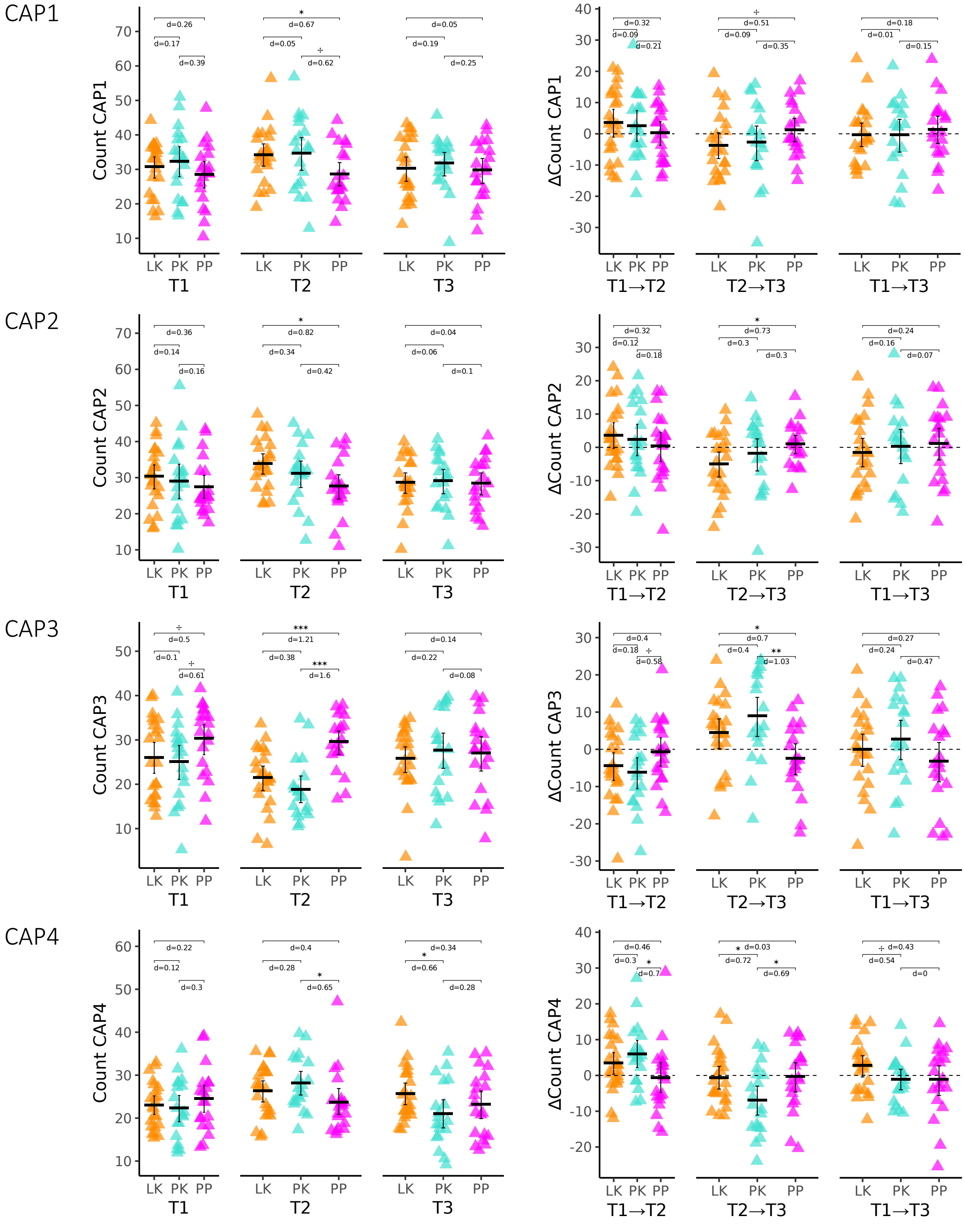

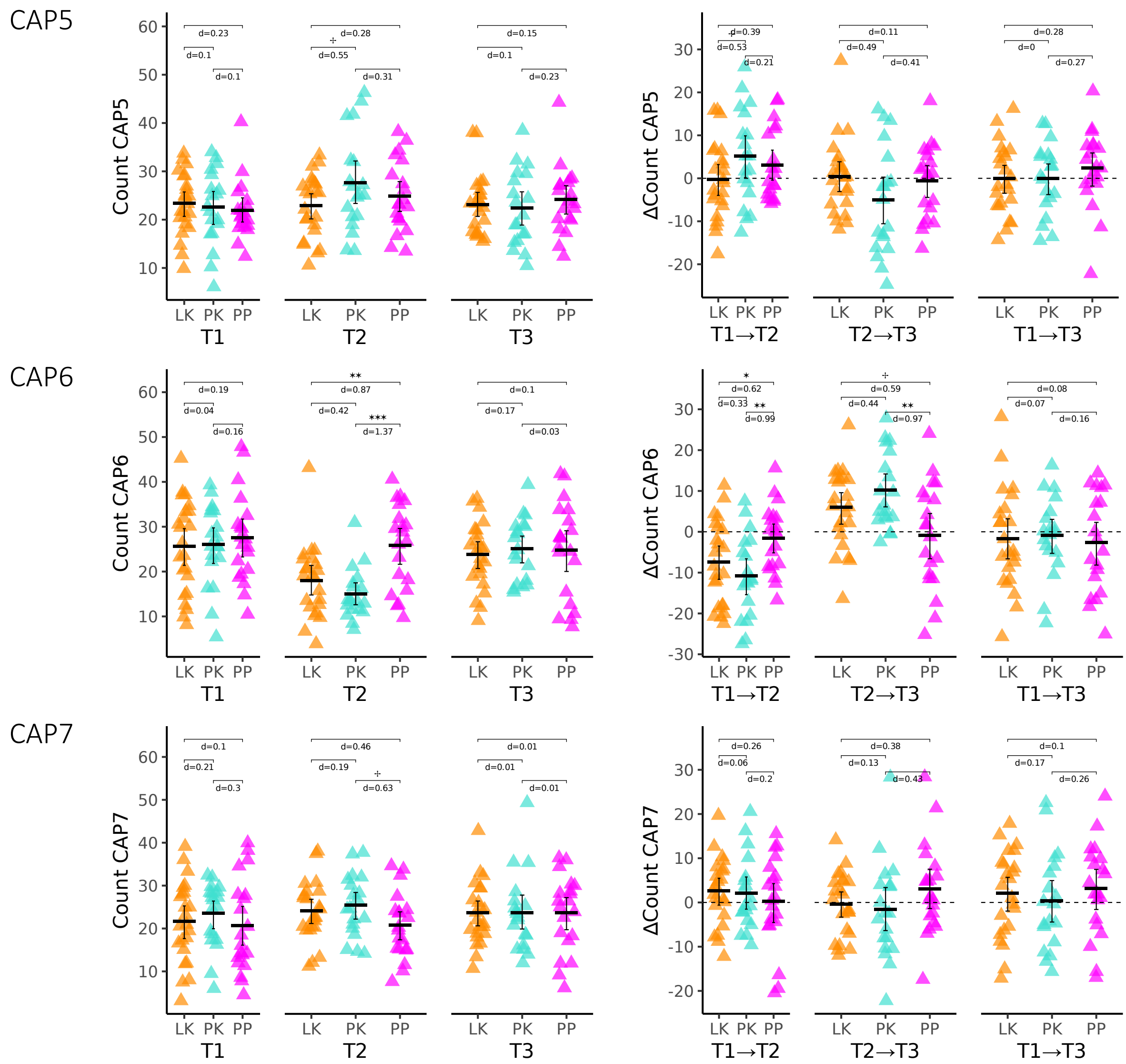

Notes. d, cohens d; +, p < .1; *, p <.05; **, p < .01; ***, p < .001; LK, lamotrigine + ketamine; PK, placebo + ketamine; PP, placebo + placebo.

Table S3. Between subject effects.

Time point	PK>PP				LK>PP				LK>PK			
	MD	*Cohens d*	*95%-CI*	*p-value*	*MD*	*Cohens d*	*95%-CI*	*p-value*	*MD*	*Cohens d*	*95%-CI*	*p-value*
CAP1												
T1	3.775	0.39	[-0.24, 1.07]	.217	2.235	0.26	[-0.32, 0.93]	.385	-1.540	-0.17	[-0.81, 0.45]	.581
T2	6.014	0.62	[-0.001, 1.37]	.054	5.514	0.67	[0.100, 1.37]	.031	-0.499	-0.05	[-0.710, 0.57]	.870
T3	2.092	0.25	[-0.36, 0.96]	.425	0.461	0.05	[-0.57, 0.65]	.857	-1.630	-0.19	[-0.88, 0.39]	.535
CAP2												
T1	1.580	0.16	[-0.48, 0.81]	.610	2.961	0.36	[-0.21, 1.12]	.229	1.381	0.14	[-0.48, 0.83]	.641
T2	3.547	0.42	[-0.20, 1.14]	.180	6.230	0.82	[0.24, 1.49]	.011	2.683	0.34	[-0.27, 0.97]	.271
T3	0.742	0.10	[-0.54, 0.79]	.757	0.258	0.04	[-0.56, 0.69]	.905	-0.484	-0.06	[-0.70, 0.55]	.830
CAP3												
T1	-5.234	-0.61	[-1.34,-0.001]	.063	-4.306	-0.50	[-1.22, 0.07]	.100	0.928	0.10	[-0.51, 0.72]	.739
T2	-10.758	-1.60	[-2.84,-0.87]	< .001	-8.091	-1.21	[-1.96,-0.63]	< .001	2.667	0.38	[-0.21, 1.18]	.224
T3	0.712	0.08	[-0.58, 0.71]	.803	-1.145	-0.14	[-0.78, 0.47]	.644	-1.857	-0.22	[-0.85, 0.42]	.474
CAP4												
T1	-2.179	-0.30	[-0.95, 0.33]	.353	-1.432	-0.22	[-0.83, 0.40]	.468	0.748	0.12	[-0.50, 0.76]	.695
T2	4.477	0.65	[0.02, 1.56]	.044	2.711	0.40	[-0.20, 1.27]	.185	-1.766	-0.28	[-0.92, 0.34]	.362
T3	-2.169	-0.28	[-0.97, 0.34]	.363	2.424	0.34	[-0.27, 0.98]	.252	4.593	0.66	[0.08, 1.36]	.038
CAP5												
T1	0.701	0.10	[-0.49, 0.84]	.747	1.454	0.23	[-0.33, 0.96]	.437	0.753	0.10	[-0.52, 0.73]	.733
T2	2.779	0.31	[-0.31, 0.94]	.330	-1.937	-0.28	[-0.88, 0.32]	.367	-4.717	-0.55	[-1.16, 0.04]	.068
T3	-1.713	-0.23	[-0.88, 0.40]	.471	-0.997	-0.15	[-0.77, 0.46]	.627	0.715	0.10	[-0.54, 0.73]	.741
CAP6												
T1	-1.503	-0.16	[-0.75, 0.52]	.635	-1.914	-0.19	[-0.79, 0.43]	.535	-0.411	-0.04	[-0.67, 0.57]	.891
T2	-10.811	-1.37	[-2.37,-0.72]	<.001	-7.819	-0.87	[-1.71,-0.27]	.006	2.992	0.42	[-0.17, 1.06]	.188
T3	0.284	0.03	[-0.62, 0.65]	.919	-0.956	-0.10	[-0.76, 0.51]	.727	-1.239	-0.17	[-0.80, 0.46]	.581
CAP7												
T1	2.860	0.30	[-0.30, 1.07]	.342	1.002	0.10	[-0.50, 0.77]	.744	-1.859	-0.21	[-0.88, 0.38]	.492
T2	4.753	0.63	[0.00, 1.37]	.051	3.392	0.46	[-0.14, 1.13]	.133	-1.360	-0.19	[-0.86, 0.43]	.540
T3	0.053	0.01	[-0.71, 0.61]	.986	-0.045	-0.01	[-0.66, 0.59]	.986	-0.097	-0.01	[-0.59, 0.65]	.974

Notes. PK, placebo + ketamine; PP, placebo + placebo; LK, lamotrigine + placebo; MD, mean difference; CI, bootstrap confidence interval.

Table S4. Group by time interactions.

	PK>PP				LK>PP				LK>PK			
Time point	*MD*	*Cohens d*	*95%-CI*	*p-value*	*MD*	*Cohens d*	*95%-CI*	*p-value*	*MD*	*Cohens d*	*95%-CI*	*p-value*
CAP1												
T1→T2	2.239	0.21	[-0.42, 0.86]	.501	3.280	0.32	[-0.28, 0.96]	.298	1.040	0.09	[-0.51, 0.75]	.763
T2→T3	-3.922	0.35	[-0.98, 0.28]	.275	-5.053	-0.51	[-1.22, 0.08]	.098	-1.131	-0.09	[-0.78, 0.48]	.763
T1→T3	-1.683	-0.15	[-0.77, 0.50]	.638	-1.774	-0.18	[-0.83, 0.42]	.560	-0.091	-0.01	[-0.68, 0.59]	.974
CAP2												
T1→T2	1.967	0.18	[-0.46, 0.83]	.571	3.269	0.32	[-0.29, 0.91]	.297	1.302	0.12	[-0.52, 0.75]	.685
T2→T3	-2.805	-0.30	[-0.89, 0.36]	.345	-5.972	-0.73	[-1.38,-0.17]	.024	-3.167	-0.30	[-1.06, 0.30]	.314
T1→T3	-0.838	-0.07	[-0.75, 0.55]	.818	-2.703	-0.24	[-0.94, 0.36]	.420	-1.865	-0.16	[-0.82, 0.46]	.596
CAP3												
T1→T2	-5.534	-0.58	[-1.25, 0.03]	.075	-3.785	-0.40	[-1.00, 0.18]	.183	1.740	0.18	[-0.45, 0.83]	.554
T2→T3	11.470	1.03	[0.40, 1.84]	.003	6.946	0.70	[0.12, 1.39]	.026	-4.524	-0.40	[-1.10, 0.21]	.195
T1→T3	5.946	0.47	[-0.16, 1.17]	.138	3.161	0.27	[-0.34, 0.91]	.382	-2.785	-0.24	[-0.90, 0.36]	.435
CAP4												
T1→T2	6.656	0.70	[0.09, 1.53]	.032	4.143	0.46	[-0.14, 1.26]	.136	-2.513	-0.30	[-0.92, 0.32]	.325
T2→T3	-6.646	-0.69	[-1.47,-0.08]	.036	-0.287	-0.03	[-0.71, 0.57]	.917	6.359	0.72	[0.15, 1.40]	.022
T1→T3	0.010	0.00	[-0.69, 0.59]	.997	3.855	0.43	[-0.16, 1.01]	.149	3.845	0.54	[-0.06, 1.23]	.089
CAP5												
T1→T2	2.078	0.21	[-0.41, 0.89]	.517	-3.391	-0.39	[-1.02, 0.21]	.211	-5.470	-0.53	[-1.23, 0.07]	.082
T2→T3	-4.492	-0.41	[-1.17, 0.22]	.204	0.940	0.11	[-0.52, 0.69]	.724	5.432	0.49	[-0.12, 1.21]	.111
T1→T3	-2.414	-0.27	[-0.99, 0.34]	.387	-2.451	-0.28	[-0.98, 0.31]	.356	-0.037	-0.00	[-0.66, 0.60]	.986
CAP6												
T1→T2	-9.308	-0.99	[-1.75,-0.39]	.004	-5.905	-0.62	[-1.30,-0.04]	.045	3.402	0.33	[-0.28, 0.97]	.279
T2→T3	11.094	0.97	[0.39, 1.66]	.004	6.863	0.59	[-0.01, 1.29]	.058	-4.231	-0.44	[-1.04, 0.16]	.164
T1→T3	1.787	0.16	[-0.48, 0.81]	.606	0.958	0.08	[-0.56, 0.69]	.803	-0.828	-0.07	[-0.74, 0.49]	.812
CAP7												
T1→T2	1.892	0.20	[-0.45, 0.79]	.527	2.390	0.26	[-0.34, 0.86]	.387	0.498	0.06	[-0.54, 0.75]	.838
T2→T3	-4.700	-0.43	[-1.15, 0.20]	.184	-3.437	-0.38	[-0.98, 0.23]	.216	1.263	0.13	[-0.46, 0.84]	.659
T1→T3	-2.808	-0.26	[-0.97, 0.38]	.422	-1.046	-0.10	[-0.72, 0.51]	.735	1.761	0.17	[-0.43, 0.85]	.592

Notes. PK, placebo + ketamine; PP, placebo + placebo; LK, lamotrigine + placebo; MD, mean difference; CI, bootstrap confidence interval.

Table S5. Within-subject effects.

	PK				LK				PP			
Time point	*MD*	*Cohens d*	*95%-CI*	*p-value*	*MD*	*Cohens d*	*95%-CI*	*p-value*	*MD*	*Cohens d*	*95%-CI*	*p-value*
CAP1												
T1→T2	2.352	0.21	[-0.24, 0.70]	.379	3.393	0.31	[-0.11,0.79]	.154	0.113	0.01	[-0.46, 0.49]	.953
T2→T3	-2.813	-0.22	[-0.68,0.27]	.366	-3.944	-0.37	[-0.96, 0.03]	.087	1.109	0.13	[-0.34, 0.58]	.578
T1→T3	-0.461	-0.04	[-0.49, 0.48]	.873	-0.552	-0.06	[-0.60, 0.32]	.787	1.222	0.12	[-0.34,0.58]	.600
CAP2												
T1→T2	2.184	0.20	[-0.25, 0.72]	.398	3.486	0.36	[-0.04, 0.78]	.098	0.217	0.02	[-0.42, 0.53]	.924
T2→T3	-2.004	-0.18	[-0.60, 0.34]	.453	-5.171	-0.55	[-1.03,-0.18]	.014	0.801	0.12	[-0.34, 0.61]	.588
T1→T3	0.180	0.02	[-0.49, 0.50]	.946	-1.685	-0.16	[-0.66, 0.25]	.456	1.018	0.09	[-0.34, 0.62]	.683
CAP3												
T1→T2	-6.299	-0.66	[-1.22,-0.26]	.010	-4.559	-0.49	[-0.96,-0.11]	.026	-0.774	-0.09	[-0.62, 0.35]	.709
T2→T3	8.895	0.73	[0.27, 1.53]	.006	4.370	0.44	[0.04, 1.03]	.049	-2.576	-0.27	[-0.73, 0.17]	.244
T1→T3	2.596	0.21	[-0.24, 0.81]	.368	-0.189	-0.02	[-0.43, 0.43]	.937	-3.350	-0.27	[-0.75, 0.17]	.244
CAP4												
T1→T2	5.838	0.68	[0.29, 1.22]	.006	3.324	0.43	[0.03, 0.92]	.050	-0.819	-0.08	[-0.68, 0.32]	.723
T2→T3	-7.100	-0.76	[-1.43,-0.33]	.003	-0.741	-0.09	[-0.62, 0.29]	.665	-0.453	-0.05	[-0.49, 0.46]	.833
T1→T3	-1.262	-0.19	[-0.77, 0.26]	.410	2.583	0.35	[-0.04, 0.81]	.113	-1.272	-0.13	[-0.53, 0.39]	.573
CAP5												
T1→T2	5.006	0.45	[0.03, 0.98]	.061	-0.464	-0.05	[-0.51, 0.36]	.800	2.928	0.36	[-0.06, 0.77]	.124
T2→T3	-5.222	-0.42	[-1.05, 0.03]	.089	0.210	0.02	[-0.48, 0.39]	.919	-0.730	-0.08	[-0.57, 0.37]	.713
T1→T3	-0.216	-0.03	[-0.50, 0.47]	.913	-0.254	-0.03	[-0.48, 0.38]	.883	2.197	0.24	[-0.18, 0.90]	.292
CAP6												
T1→T2	-11.023	-1.09	[-1.84,-0.65]	<.001	-7.620	-0.74	[-1.30,-0.35]	.002	-1.715	-0.21	[-0.73, 0.24]	.363
T2→T3	10.019	1.09	[0.77, 1.59]	<.001	5.789	0.60	[0.20, 1.22]	.010	-1.075	-0.08	[-0.55, 0.38]	.725
T1→T3	-1.003	-0.10	[-0.56, 0.39]	.663	-1.832	-0.15	[-0.65, 0.26]	.485	-2.790	-0.23	[-0.72, 0.22]	.306
CAP7												
T1→T2	1.942	0.24	[-0.25, 0.66]	.327	2.440	0.32	[-0.08, 0.81]	.141	0.050	0.00	[-0.42, 0.54]	.983
T2→T3	-1.775	-0.16	[-0.78, 0.28]	.494	-0.512	-0.07	[-0.50, 0.35]	.732	2.925	0.28	[-0.15, 0.72]	.229
T1→T3	0.167	0.02	[-0.52, 0.46]	.945	1.928	0.20	[-0.20, 0.69]	.351	2.974	0.28	[-0.16, 0.85]	.220

Notes. PK, placebo + ketamine; PP, placebo + placebo; LK, lamotrigine + placebo; MD, mean difference; CI, bootstrap confidence interval.

Additional information

Here, additional information regarding sample size determination, randomization, blinding, adverse events, physical examination and plasma concentration of lamotrigine and ketamine are provided. Please note that a majority of the following information are already provided in previous publications (Gärtner et al., 2024; Gärtner, Weigand, Keicher, et al., 2023; Gärtner, Weigand, Meiering, et al., 2023; Meiering et al., 2024; Weigner et al., 2025).

Sample Size

Power analysis for a one-way ANOVA with 3 groups was conducted in G*Power (Faul et al., 2007) to determine a sufficient sample size using an alpha of 0.05, a power of 0.80, and a large effect size of f = 0.4. This effect size (averaged for acute and delayed effects of ketamine as both are primary endpoints) was based on previously reported effect sizes (d = 0.815 for acute effects and d = 0.776 for delayed ketamine effects, (Abdallah et al., 2018)), showing a significant increase in prefrontal global connectivity during infusion and at 24-h posttreatment as compared to placebo. The sample size needed with this effect size was N = 66 (22 subjects per group). Accounting for a drop-out rate of ∼15 %, the estimated total sample size was N = 75 (25 subjects per group). Screening for the trial was stopped once a sufficient number of subjects had been measured and controlled for dropouts due to excessive head movement in the scanner and/or headaches. Total study duration was from March 05, 2020 to December 10, 2020.

Randomization

Randomization, enrollment of participants, and assignment to interventions was conducted by the Charité Research Organisation (CRO), GmbH. After written informed consent, subjects meeting all in-/exclusion criteria were randomized at baseline to one of three experimental conditions (lamotrigine + ketamine, placebo + ketamine, placebo). The three treatments were allocated 1:1:1 randomization. Each subject received only one single dose of blinded combined study medication (lamotrigine + ketamine, placebo + ketamine or placebo) in the sequence according to randomization administered by the site staff. Randomization numbers were assigned in ascending, sequential order to eligible subjects. Additional numbers were used in case of replacements being needed. The investigator documented the randomization number

in the eCRF. The randomization list was kept in safe and confidential custody. Only personnel not involved in the study had access to the list.

Blinding

For the conduct of this study, the study drugs as well as matching placebos (lamotrigine + ketamine, placebo + ketamine or placebo) were administered in a double-blind fashion at the site. Doses were prepared by an unblinded member of the study team. This unblinded member was not involved in any study assessments. The identity of the treatments was concealed by the use of study drugs that were all identical in packaging, labeling, schedule of administration, appearance, and odor. Only the unblinded site team had access to the unblinded randomization list. Subjects remained blinded to study treatment throughout the study. Anyone who was involved in subject-related assessments was blinded with regards to the treatment assigned.

Harms

Safety assessments included vital signs, physical examinations, ECGs, standard clinical laboratory evaluations (hematology, blood chemistry, and coagulation), adverse events and serious adverse event monitoring.

Adverse events

No severe adverse events occurred. At screening appointments, a total of n = 3 minor pretreatment adverse events were noted: Respectively one participant was suffering from a skin rash, herpes labialis and a hordeolum.

Vital signs

Vital signs included pulse rate, systolic and diastolic blood pressure and body temperature. The body temperature was measured in the ear. Vital signs could be recorded at any time, if medically imperative for clarification of clinical signs and symptoms. For n = 35 participants abnormal vital signs were reported; body temperature, blood pressure or pulse rate were out of range. None of the abnormal vital signs were clinically significant, except for one participant who, however, reported to feel well. During the course of the study, none of the participants was excluded due to abnormal vital signs. A detailed description of all abnormal vital signs can be presented on request.

Headache severity

A total of n = 16 participants reported headaches either prior or after fMRI assessment, mean NRS score was = 1.4, all NRS scores were < 5, thus none of the participants was excluded due to reported headache.

Electrocardiogram

For n = 3 participants abnormal ECG values were detected. In accordance with the study protocol, measurements were repeated. During the repeated measurements, no abnormal values were detected.

Physical examination

Information for all physical examinations was included in the source documentation at the study site, only medically relevant findings were recorded in the eCRF. For examinations at the Screening visit, medically relevant findings were also captured as medical history in the eCRF.

Deaths and other serious adverse events

No AEs leading to discontinuation or other significant AEs occurred during the study.

Adverse Events leading to discontinuation and other significant AEs

One subject discontinued study participation due to a panic reaction in the scanner.

Clinical laboratory evaluation

The following parameters were assessed: sodium, postassium, calcium, magnesium, total protein, albumin, glucose, creatinine, urea, bilirubin, AST, ALT, GGT, LDH, AP, CRP; hematology: leukocytes, granulocytes, neutrophils, eosinophils, basophiles, lymphocytes, monocytes, erythrocytes, thrombocytes, haematocrit, haemoglobin; coagulation: aPTT, INR.

Plasma concentration

Plasma concentrations for lamotrigine and ketamine are depicted in the table below. No significant differences in ketamine plasma concentration was found between the lamotrigine

and ketamine group (T(44) = 1.62, p = 0.11). These results were already published by Gärtner et al. 2023.

Table S3.

Ketamine	
+0:55 h	PK: 108.59 ng/L ± 27.61<br>LK: 94.26 ng/L ± 32.45
Lamotrigine	
+0:30 h	2998.17 mg/L ± 1316.80
+1:00 h	3509.76 mg/L ± 1153.81
+1:30 h	3986.10 mg/L ± 990.62
+2:55 h	3943.42 mg/L ± 928.82
+4:00 h	3791.87 mg/L ± 698.83

Notes: Time points refer to infusion onset for ketamine and oral administration for lamotrigine.

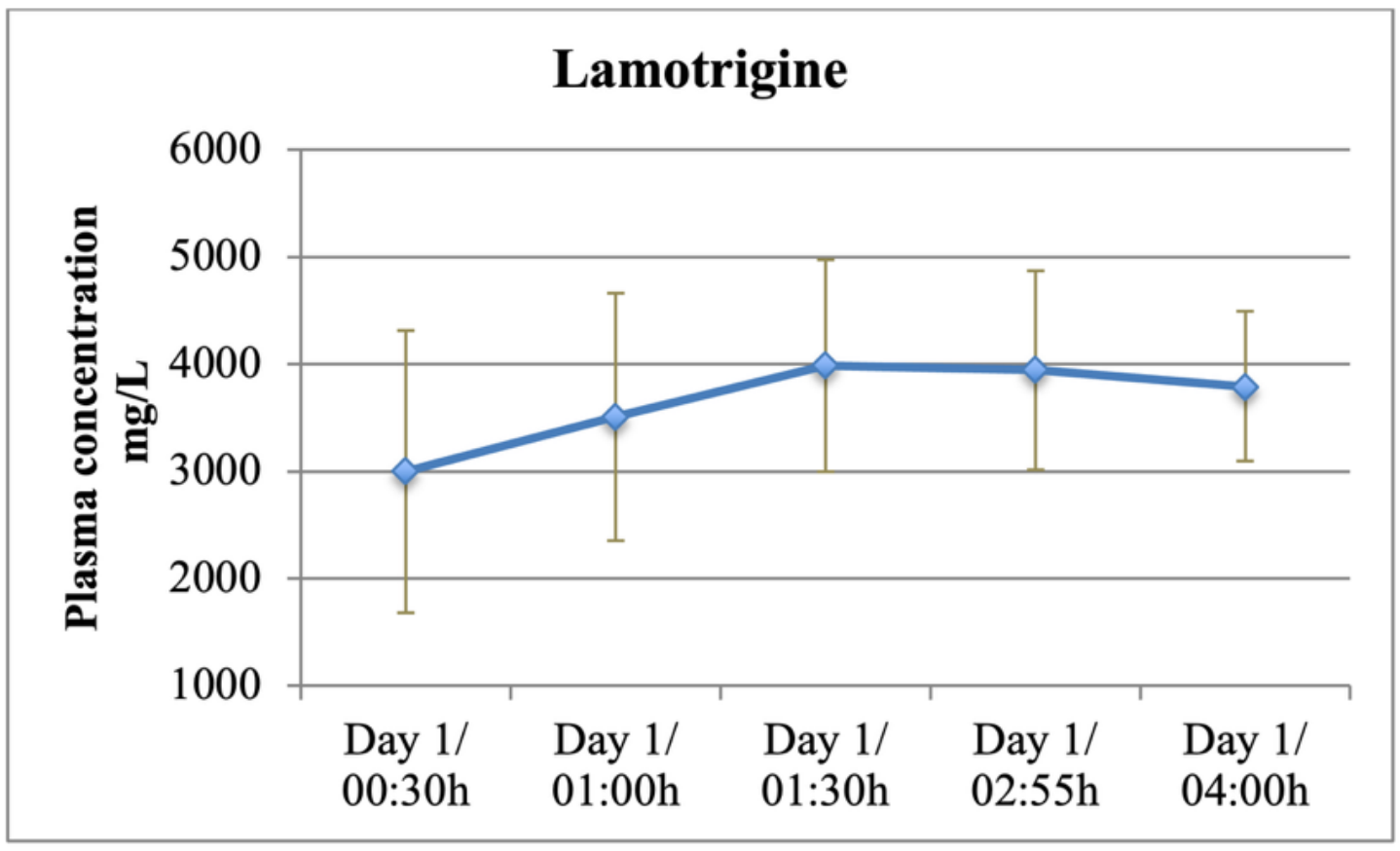

Figure S6. Plasma concentration of lamotrigine. Error bars depicting standard deviations. Time points refer to lamotrigine administration.

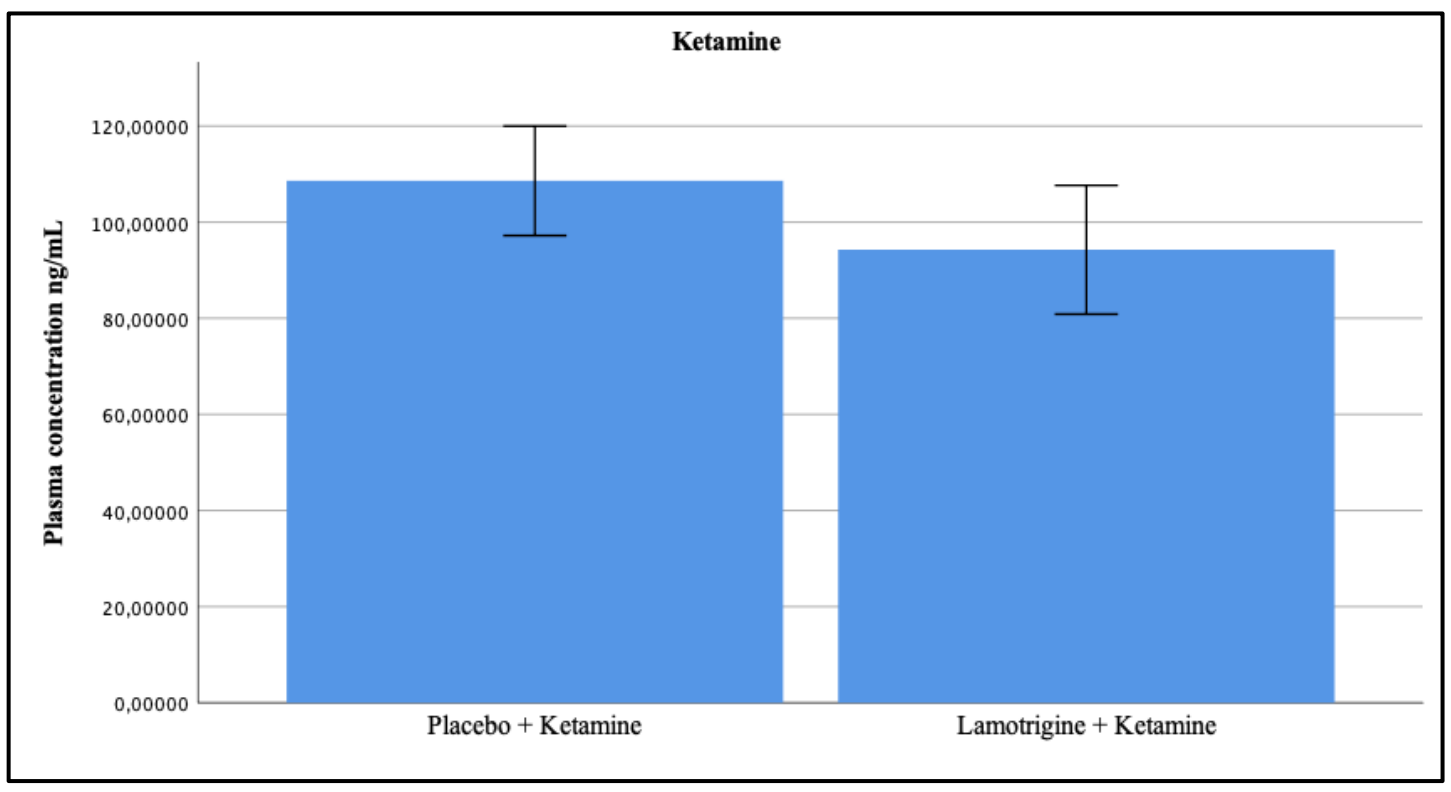

Figure S7. Plasma concentration of ketamine. Error bars depicting 95% confidence intervals.

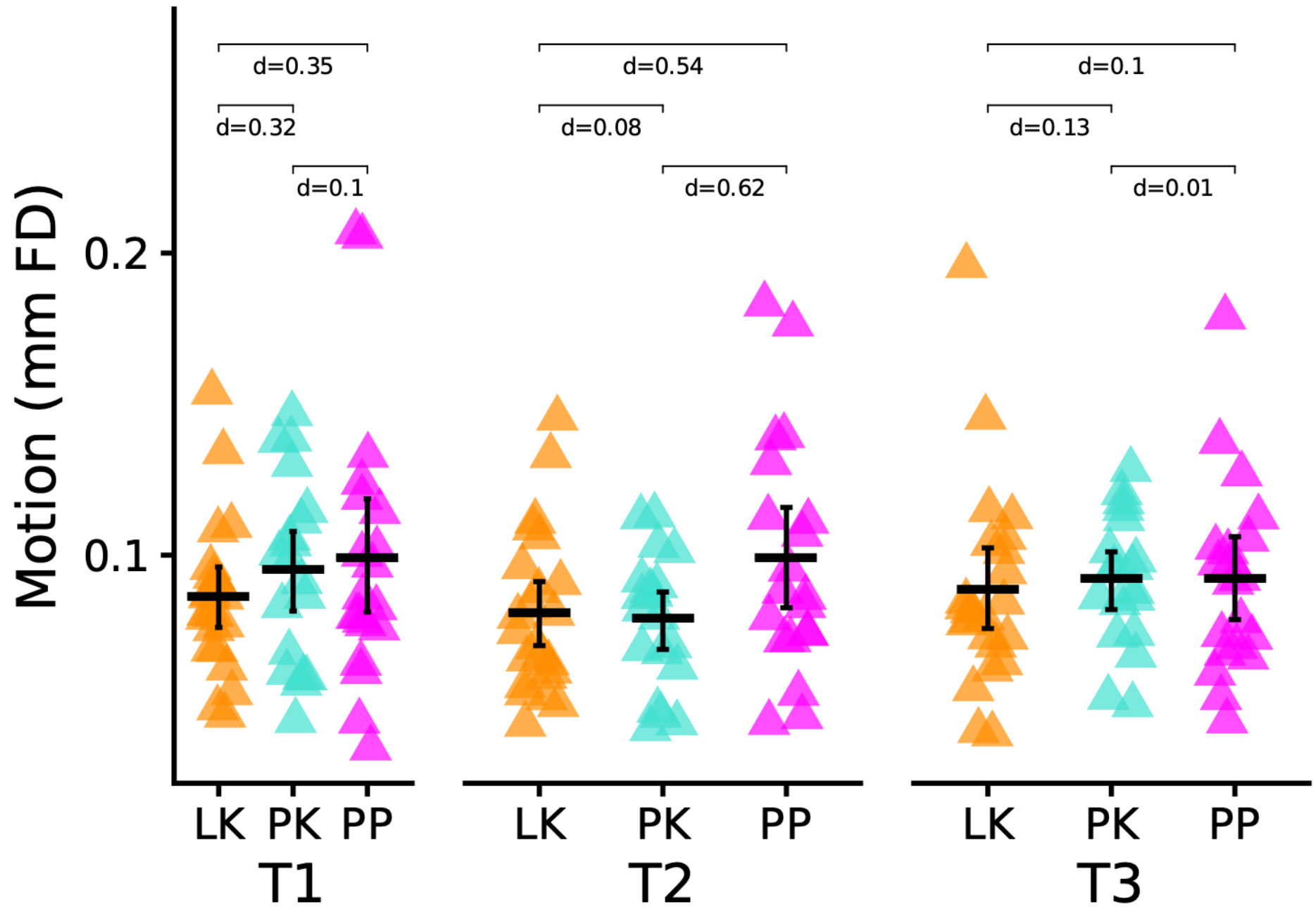

Figure S8. Motion. FD, framewise displacement in mm; d, Cohens d; LK, lamotrigine + ketamine; PK, placebo + ketamine; PP, placebo + placebo.

References

Abdallah, C. G., Sanacora, G., Duman, R. S., & Krystal, J. H. (2018). The neurobiology of depression, ketamine and rapid-acting antidepressants: Is it glutamate inhibition or activation? *Pharmacology & Therapeutics*, *190*, 148–158. https://doi.org/10.1016/j.pharmthera.2018.05.010

Beckmann, C. F., & Smith, S. M. (2004). Probabilistic Independent Component Analysis for Functional Magnetic Resonance Imaging. *IEEE Transactions on Medical Imaging*, *23*(2), 137–152. https://doi.org/10.1109/TMI.2003.822821

Beckmann, C. F., & Smith, S. M. (2005). Tensorial extensions of independent component analysis for multisubject FMRI analysis. *NeuroImage*, *25*(1), 294–311. https://doi.org/10.1016/j.neuroimage.2004.10.043

Faul, F., Erdfelder, E., Lang, A.-G., & Buchner, A. (2007). G*Power 3: A flexible statistical power analysis program for the social, behavioral, and biomedical sciences. *Behavior Research Methods*, *39*(2), Article 2. https://doi.org/10.3758/BF03193146

Gärtner, M., Weigand, A., Keicher, C., Meiering, M. S., Weigner, D., Carstens, L., Hertrampf, R., Beckmann, C., Mennes, M., Wunder, A., & Grimm, S. (2023). Modulatory Effects of Ketamine and Lamotrigine on Cognition: Emotion Interaction in the Brain. *Neuropsychobiology*, *82*(2), 91–103. https://doi.org/10.1159/000528315

Gärtner, M., Weigand, A., Meiering, M. S., Weigner, D., Carstens, L., Keicher, C., Hertrampf, R., Beckmann, C., Mennes, M., Wunder, A., & Grimm, S. (2023). Region- and time- specific effects of ketamine on cerebral blood flow: A randomized controlled trial. *Neuropsychopharmacology*, *48*, 1–7. https://doi.org/10.1038/s41386-023-01605-4

Gärtner, M., Weigand, A., Meiering, M. S., Weigner, D., Carstens, L., Keicher, C., Hertrampf, R., Beckmann, C., Mennes, M., Wunder, A., & Grimm, S. (2024). Negative emotionality

shapes the modulatory effects of ketamine and lamotrigine in subregions of the anterior cingulate cortex. *Translational Psychiatry*, *14*(258), 2–7. https://doi.org/10.1038/s41398-024-02977-x

Jenkinson, M., Beckmann, C. F., Behrens, T. E. J., Woolrich, M. W., & Smith, S. M. (2012). FSL. *NeuroImage*, *62*(2), 782–790. https://doi.org/10.1016/j.neuroimage.2011.09.015

Meiering, M. S., Weigner, D., Gärtner, M., Carstens, L., Keicher, C., Hertrampf, R., Beckmann, C. F., Mennes, M., Wunder, A., Weigand, A., & Grimm, S. (2024). Functional activity and connectivity signatures of ketamine and lamotrigine during negative emotional processing: A double-blind randomized controlled fMRI study. *Translational Psychiatry*, *14*(1), 436. https://doi.org/10.1038/s41398-024-03120-6

Pruim, R. H. R., Mennes, M., Buitelaar, J. K., & Beckmann, C. F. (2015). Evaluation of ICA-AROMA and alternative strategies for motion artifact removal in resting state fMRI. *NeuroImage*, *112*, 278–287. https://doi.org/10.1016/j.neuroimage.2015.02.063

Pruim, R. H. R., Mennes, M., Van Rooij, D., Llera, A., Buitelaar, J. K., & Beckmann, C. F. (2015). ICA-AROMA: A robust ICA-based strategy for removing motion artifacts from fMRI data. *NeuroImage*, *112*, 267–277. https://doi.org/10.1016/j.neuroimage.2015.02.064

Smith, S. M., Jenkinson, M., Woolrich, M. W., Beckmann, C. F., Behrens, T. E. J., Johansen-Berg, H., Bannister, P. R., De Luca, M., Drobnjak, I., Flitney, D. E., Niazy, R. K., Saunders, J., Vickers, J., Zhang, Y., De Stefano, N., Brady, J. M., & Matthews, P. M. (2004). Advances in functional and structural MR image analysis and implementation as FSL. *NeuroImage*, *23*, S208–S219. https://doi.org/10.1016/j.neuroimage.2004.07.051

Weigner, D., Meiering, M. S., Weigand, A., Carstens, L., Keicher, C., Hertrampf, R., Beckmann, C., Mennes, M., Wunder, A., Gärtner, M., & Grimm, S. (2025). Functional connectivity alterations of the pregenual anterior cingulate cortex by ketamine and the modulation

by lamotrigine. *Journal of Psychopharmacology*, 02698811251346705. https://doi.org/10.1177/02698811251346705

Woolrich, M. W., Behrens, T. E. J., Beckmann, C. F., Jenkinson, M., & Smith, S. M. (2004). Multilevel linear modelling for FMRI group analysis using Bayesian inference. *NeuroImage*, *21*(4), 1732–1747. https://doi.org/10.1016/j.neuroimage.2003.12.023

Woolrich, M. W., Behrens, T. E. J., & Smith, S. M. (2004). Constrained linear basis sets for HRF modelling using Variational Bayes. *NeuroImage*, *21*(4), 1748–1761. https://doi.org/10.1016/j.neuroimage.2003.12.024

Woolrich, M. W., Jbabdi, S., Patenaude, B., Chappell, M., Makni, S., Behrens, T., Beckmann, C., Jenkinson, M., & Smith, S. M. (2009). Bayesian analysis of neuroimaging data in FSL. *NeuroImage*, *45*(1), S173–S186. https://doi.org/10.1016/j.neuroimage.2008.10.055

Woolrich, M. W., Ripley, B. D., Brady, M., & Smith, S. M. (2001). Temporal Autocorrelation in Univariate Linear Modeling of FMRI Data. *NeuroImage*, *14*(6), 1370–1386. https://doi.org/10.1006/nimg.2001.0931
